# Supplementary material for: Enantiomeric glycosylated cationic block co-beta-peptides eradicate Staphylococcus aureus biofilms and antibiotic-tolerant persisters
Source: Nat Commun. 2019 Oct 21;10:4792. doi: 10.1038/s41467-019-12702-8 (PMC6803644; doi:10.1038/s41467-019-12702-8)
Supplement: Supplementary file 1 — Supplementary Information [file 41467_2019_12702_MOESM1_ESM.pdf]

Supplementary Information

Enantiomeric glycosylated cationic block co-beta-peptides eradicate *Staphylococcus aureus* biofilms and antibiotic-tolerant persisters

Zhang *et al.*

Supplementary Methods

General Methods and Instrumentation

Commercially available materials purchased from Alfa Aesar or Aldrich were used as received. All reactions were carried out under argon using standard techniques, unless otherwise noted. THF was distilled over sodium benzophenone ketyl under argon. Proton nuclear magnetic resonance (^{1}H NMR) spectra were recorded on a Bruker AV400 (400 MHz) spectrometer. Chemical shifts were recorded in parts per million (ppm, δ) relative to tetramethylsilane (δ 0.00) in $CDCl_3$, or HOD (δ 4.79) in D_2O. Carbon nuclear magnetic resonance (^{13}C NMR) spectra were recorded on a Bruker AV400 (100 MHz) spectrometer. Protected polymer molecular weights were determined by gel permeation chromatography (GPC) versus polystyrene standards using DMF (1 mg mL^{-1} LiBr) as the eluent at a flow rate of 1.0 mL min^{-1} through two Styragel columns (HR5 and HR5E, 7.8 × 300 mm) in series at 40 ºC with a refractive index detector. Circular dichroism (CD) spectra were obtained using a Chirascan circular dichroism spectrometer with samples dissolved in buffer in a 1 cm path-length quartz cuvette. Flash chromatography was performed using Merck silica gel 60 with distilled solvents. Analytical thin-layer chromatography (TLC) was carried out on Merck 60 F254 pre-coated silica gel plate (0.2 mm thickness). Visualization was performed using a UV lamp. Matrix-Assisted Laser Desorption/Ionization-Time of Flight (MALDI-TOF) measurements were obtained using Applied Biosystems 4700 series. Biomarkers of in vivo studies were recorded using Blood Chemistry Analyzer Pointcare V2 (MNChip).

Monomer Synthesis Procedures

Synthesis of *N*-Cbz-*β*-lactam-L-hLys 1 (**BLK$_p$**)

β^3-hLys was prepared following a published procedure[1]. Starting from a commercially available protected form of amino acid L-lysine, β^3-hLys was obtained in high yield via Arndt-Eistert reaction. Then β^3-hLys was cyclized following the general procedure of Mukaiyama[2] to generate *N*-Boc-*β*-lactam-L-hLys. A solution of *N*-Boc-*β*-lactam-L-hLys (970 mg, 4 mmol) in dichloromethane (5 mL) was added to trifluoroacetic acid (3.1 mL, 40 mmol) dropwise at 0 ºC, the mixture was stirred at room temperature for about 1 hour until the *N*-Boc-*β*-lactam-L-hLys was completely consumed (monitored

by TLC). After completion, the mixture was cooled to 0 ºC, then quenched with a saturated aqueous solution of $NaHCO_3$ (20 mL). The reaction was diluted with THF (10 mL) followed by the addition of benzyl chloroformate (0.7 mL, 5 mmol), the resulting mixture was stirred at room temperature for 8 hours and then extracted with diethyl ether (2 × 20 mL). The combined organic extracts were then dried over anhydrous Na_2SO_4, filtered and concentrated under reduced pressure. The crude residue was purified by flash column chromatography (5:1 EtOAc:hexane) on silica gel and then recrystallized from EtOAc/hexane to give *N*-Cbz-*β*-lactam-L-hLys **1** (**BLK$_p$**) as a white solid (yield > 90%). R_f 0.25, (eluent: EtOAc:hexane = 5:1); ^{1}H NMR (400 MHz, $CDCl_3$) δ 1.33 (m, 2H), 1.45-1.68 (m, 4H), 2.53 (d, J = 14.7 Hz, 1H), 3.02 (dd, J = 14.7, 3.0 Hz, 1H), 3.19 (dd, J = 12.8, 6.4 Hz, 2H), 3.56 (m, 1H), 4.90-5.25 (3H), 6.38 (s, 1H), 7.25-7.45 (m, 5H); ^{13}C NMR (100 MHz, $CDCl_3$) δ 23.3, 29.6, 34.9, 40.7, 43.3, 47.9, 66.6, 128.05, 128.07, 128.5, 136.5, 156.4, 168.2; HRMS (ESI, *m/z*) calcd. for $C_{15}H_{20}N_2O_3H^+$: 277.1547, found: 277.1548.

Synthesis of *O*-Bn-*β*-lactam-D-glucose 2 (**DGu$_p$**)

The cyclic sugar derived *β*-lactam monomer **2** (*O*-Bn-*β*-lactam-D-glucose or **DGu$_p$**) was prepared on multigram scales in moderate yield following reported methods[3] via the stereoselective cycloaddition of tri-*O*-benzyl-D-glucal and chlorosulfonyl isocyanate, followed by *in situ* reduction to remove the sulfonyl group.

General Polymerization Procedure

Stock solutions of the monomers **1** and **2** were prepared in a glove box by weighing 2 mmol of each monomer separately into oven-dried 10 mL volumetric flasks. The lactams were dissolved in anhydrous THF and diluted to the mark ([monomer] = 0.20 M). The stock solution of 4-*t*-butylbenzoyl chloride (*t*BuBzCl) was prepared by weighing 100.3 mg *t*BuBzCl (0.50 mmol, 98% pure) into a 25 mL volumetric flask and diluting to the mark with THF ([*t*BuBzCl] = 0.02 M). The stock solution of lithium bis(trimethylsilyl)amide (LiHMDS) was prepared by weighing 215.6 mg LHMDS (1.25 mmol, 97% pure) into a 25 mL volumetric flask and diluting to the mark with THF ([LHMDS] = 0.05 M).

Into an oven-dried Schlenk tube equipped with a magnetic stir bar was placed a total of 2 mL (0.4 mmol) of monomer solution, adjusted for the desired proportion of each monomer in the polymerization feed (*e.g.*, for **PDGu$_p$(10)-*b*-PBLK$_p$(10)**, a 1:1 mixture

of **1** and **2**, 1 mL of **1** and 1 mL of **2** stock solutions was used). To the mixture was then added 1 mL (0.02 mmol, 5 mol %) of *t*BuBzCl stock solution. The Schlenk tube was sealed, removed from the glove box and cooled to -30 ºC under argon atmosphere. To the stirring reaction solution was then added 1 mL (0.05 mmol, 12.5 mol %) of LHMDS stock solution. The resulting mixture was stirred at −30 ºC for about 8 hours until the reaction finished (monitored by TLC), and was then quenched with methanol. After completion, the solution was transferred into a plastic 50 mL centrifuge tube, and the reaction vial was rinsed with a small amount of THF such that the total tube volume was about 5 mL. Hexane (40 mL) was then added to the tube, from which a white solid precipitated. The mixture was centrifuged, and the supernatant solution was decanted. After two more repetitions of the precipitation/centrifugation procedure, the white pellet was dried overnight under a nitrogen stream to yield the protected product **PDGu$_p$(x)-*b*-PBLK$_p$(y)** as a white powder. 1 mg of the sample was retained for characterization by GPC (Figure 1f and Supplementary Table 2). ^{1}H NMR spectra of **PDGu$_p$(x)-*b*-PBLK$_p$(y)** are shown in Supplementary Figure 6.

Debenzylation of Polymers

Polymer **PDGu$_p$(10)-*b*-PBLK$_p$(10)** (145 mg) and 54 mg (0.48 mmol, approx. 1.2 equiv. to monomers) of potassium *tert*-butoxide (KO*t*-Bu) were dissolved in 5.0 mL of tetrahydrofuran. The polymer solution was added dropwise to a rapidly stirred solution of sodium (160 mg, 7.0 mmol) in liquid ammonia (15 mL) at -78 ºC under nitrogen. Sodium was washed in toluene and hexane and cut into small pieces before addition. The reaction mixture was warmed to -55 °C and maintained at this temperature for about 2 hours, after which a saturated aqueous solution of ammonium chloride (NH_4Cl, 10 mL) was added to quench the reaction. Meanwhile, the deep blue color disappeared. The solution was warmed to room temperature in a water bath to evaporate the ammonia. The resulting clear solution was filtered, washed with DI water and dialyzed with 1,000 MWCO tubing for 36 hours with 10 water changes. After lyophilization, **PDGu(7)-*b*-PBLK(13)** was obtained as an amorphous white solid (>90% yield). Other copolymers (**PDGu(x)-*b*-PBLK(y),** x+y=20) were synthesized using the same conditions. NMR integrations showed that the ratio of **DGu** to **BLK** in **PDGu(x)-*b*-PBLK(y)** differed from the stoichiometric ratio of added monomers **1** and **2** in the polymerization step (Table 1, Supplementary Figure 7).

Molecular weight determination using MALDI-TOF

The molecular weights of (a) homocationic homopolymer **PBLK(20)**, (b) homosugar **PDGu(20)** and (c) block copolymer **PDGu(7)-*b*-PBLK(13)** were determined using Matrix Assisted Laser Desorption/Ionization-Time of Flight (MALDI-TOF) using α-Cyano-4-hydroxycinnamic acid (CHCA) as the matrix. Signals were obtained in positive ion mode at polymer concentration of 10 mg mL^{-1}. The mass spectrometry was analyzed using a MATLAB® based data analysis workflow with functions provided in Bioinformatics Toolbox™. The calculated molecular weights Mn were 3012 Da, 3159 Da and 3391 Da for homocationic, homosguar and block copolymer respectively.

Membrane assays

Propidium iodide (PI) (L13152 Invitrogen) was used following the manufacturers' protocol. Log phase MRSA USA300 was grown as previously described and washed three times with PBS, and resuspended to a final concentration of 10^8 CFU mL^{-1} in PBS. Polymers were added to bacteria suspension to achieve desired concentrations and incubated for 30 minutes or 1.5 hours before washing and staining with PI dye for 15 minutes. Samples were washed twice, diluted to 10^7 CFU mL^{-1} in PBS and analyzed using Flow cytometry (BD Accuri C6 plus). Data were plotted as histogram of FL3-A channel. Bacteria without polymer treatment, dead bacteria (70°C, 30min) and nisin treated bacteria (128 µg mL^{-1}) served as negative control, positive control and antibiotics control respectively. Data gating and analysis was done using FCS Express 6 plus.

Cytoplasmic membrane depolarization of the polymer was determined using the membrane potential-sensitive dye $DiSC_35$. Bacteria cells in mid-log phase were centrifuged and washed twice using 5mM HEPES buffer (pH 7.8) containing 20mM Glucose and 0.1M KCl. The bacteria were resuspended in the same buffer and diluted to a final concentration of 10^7 CFU mL^{-1}. $DiSC_35$ solution was added to bacteria suspension to achieve a final concentration of 100nM. $DiSC_35$ dye was gradually quenched at room temperature for 30 minutes before added to a black 96 well plate (Costar). Polymer solution was added to achieve desired concentration in the 96 well plate. Gramicidin S (8 µg mL^{-1}) was used as positive control. Changes in fluorescence

due to the disruption of the membrane potential gradient ($\Delta\psi$) across the cytoplasmic membrane were recorded 5 minutes after polymer addition with Tecan reader at an excitation wavelength of 622 nm and an emission wavelength of 670 nm. Errors bar were produced from triplicate measurements. Data were presented as folds fluorescent intensity change *vs* untreated bacteria control. Gramicidin S control resulted in 8.5 folds change of intensity.

Secondary structure studies

For Circular dichroism study, polymer was dissolved at 0.05 mg mL^{-1} in DI water, 10 mM phosphate buffer (pH 2.6-8.7), 20 mM carbonate buffer (pH 10.8) and in the presence of POPG liposomes (model bacterial membrane) and POPC liposomes (model mammalian membrane). Circular dichroism spectra were measured from 190 nm to 260 nm with 0.5 nm step size, with each measurement performed twice. The final data are presented as the mean value after background extraction.

Liposomes were prepared by dissolving POPG (2-oleoyl-1-palmitoyl-sn-glycero-3-phospho-rac-(1-glycerol), Avanti company) or POPC (2-oleoyl-1-palmitoyl-sn-glycero-3-phosphocholine, Avanti company) in chloroform at 10 mg mL^{-1} and vacuum drying at room temperature for 30 minutes. The lipid thin film was rehydrated with DI water, sonicated in hot water bath for 2 minutes, followed by vigorous vortexing for 2 minutes. The sonication-vortexing process was repeated 3 more times and the lipid suspension was extruded manually using a Mini-Extruder (Avanti®) through a 100nm membrane to obtain unilamellar liposomes.

Supplementary Figures

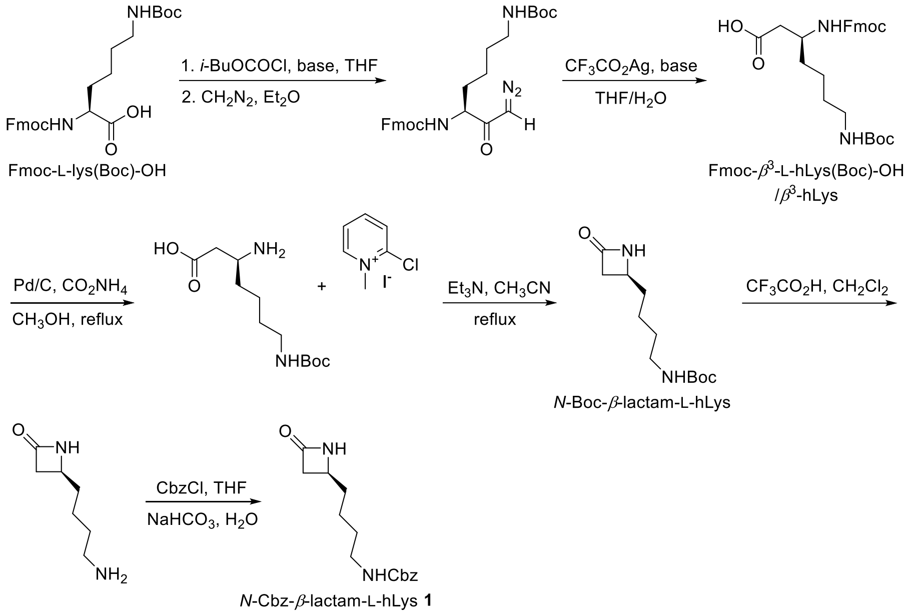

Supplementary Figure 1 Synthesis scheme of monomer *N*-Cbz-*β*-lactam-L-hLys (**BLK$_{\mathbf{p}}$**).

a

b

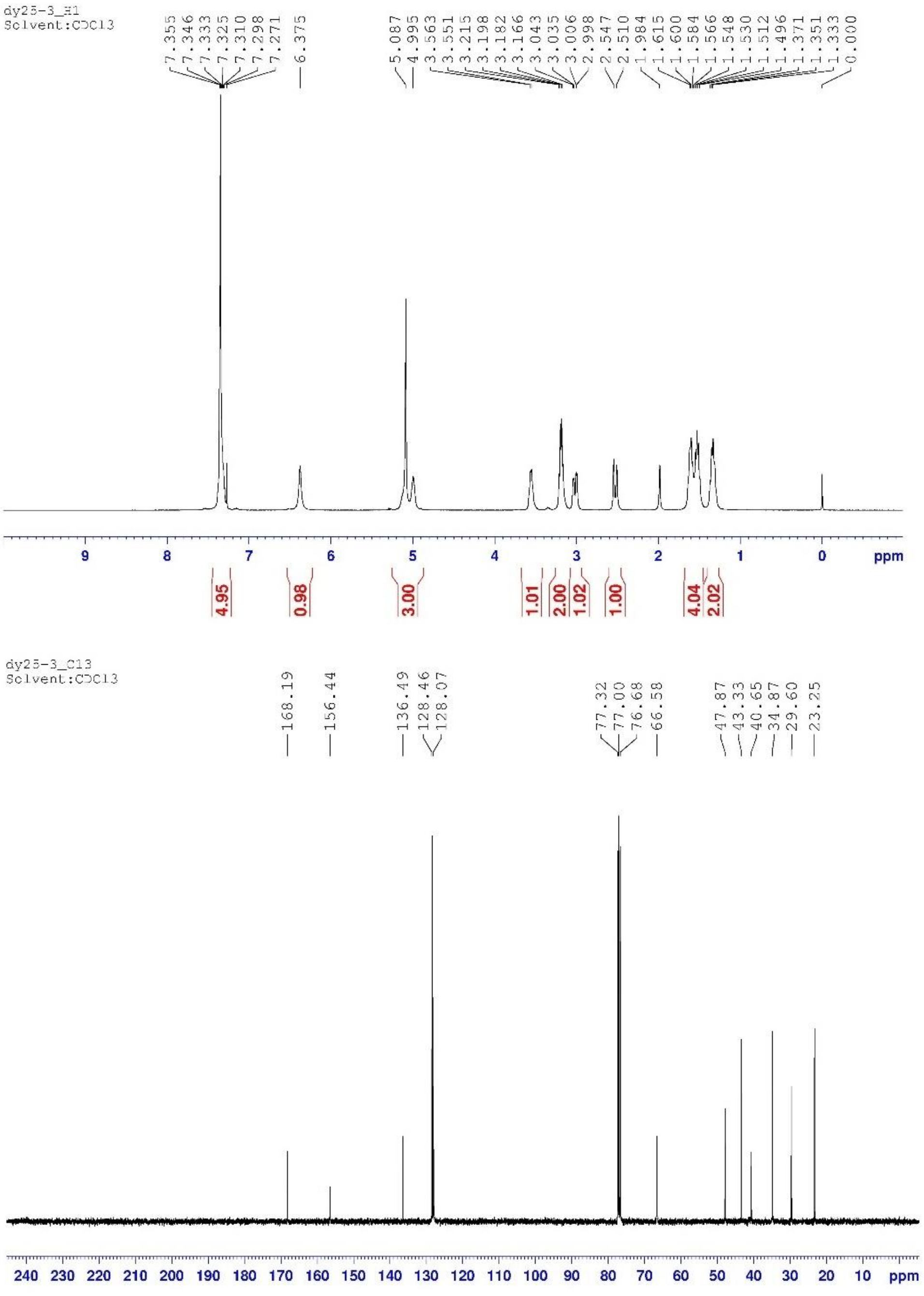

Supplementary Figure 2 NMR spectra of **BLK_p**. **(a)** ^{1}H & **(b)** ^{13}C

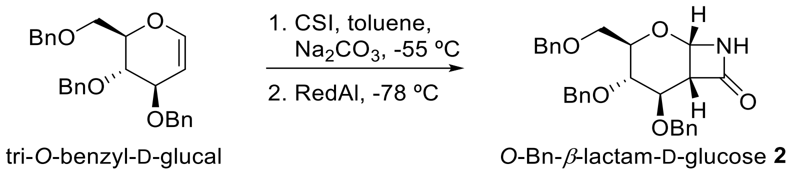

Supplementary Figure 3 Synthesis scheme of monomer *O*-Bn-*β*-lactam-D-glucose (**DGu$_p$**).

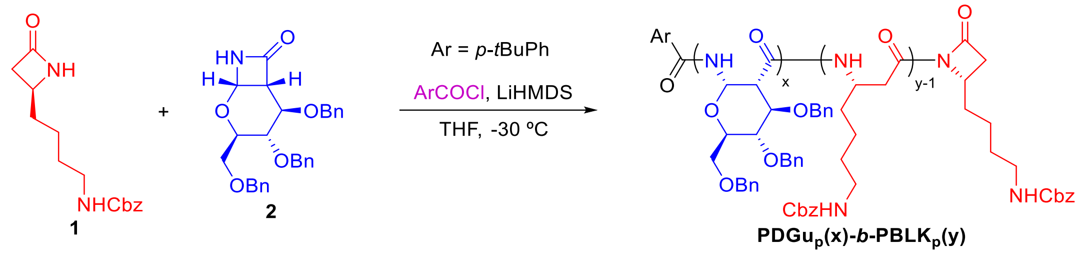

Supplementary Figure 4 Synthesis scheme of protected block copolymers **PDGu$_p$(x)-b-PBLK$_p$(y)**.

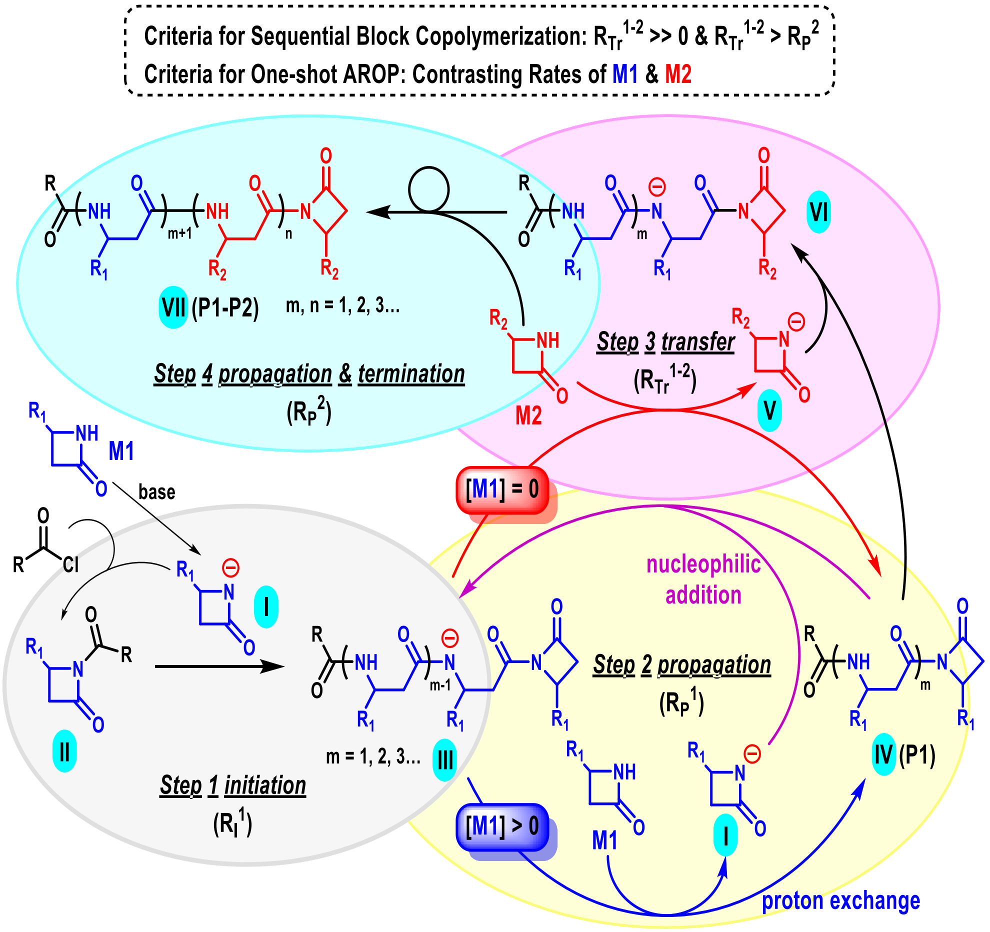

Supplementary Figure 5 Synthesis mechanism of block copolymers by AROP. Before the initiation of the polymerization, the beta-lactam (M1) is deprotonated by a base (LiHMDS) to obtain the beta-lactamate anion I; simultaneously, the beta-lactam is promoted to form an imide II. The initiation then involves the reaction of the anionic monomer I with the imide II to form a dimeric anion III (Step 1). Proton transfer exchange then occurs between the anion III and another beta-lactam (M1) to form a new imide IV and another beta-lactamate anion I respectively. Propagation is repeated nucleophilic addition of the beta-lactamate anion I to imide IV followed by proton transfer (Step 2). After M1 is consumed, M2 is initiated with the active terminal of anion III with proton transfer followed by nucleophilic addition of a new generated beta-lactamate anion V to form an anionic intermediate VI (Step 3), which undergoes propagation with M2 and final termination (Step 4) to form the block copolymer VII (P1-P2).

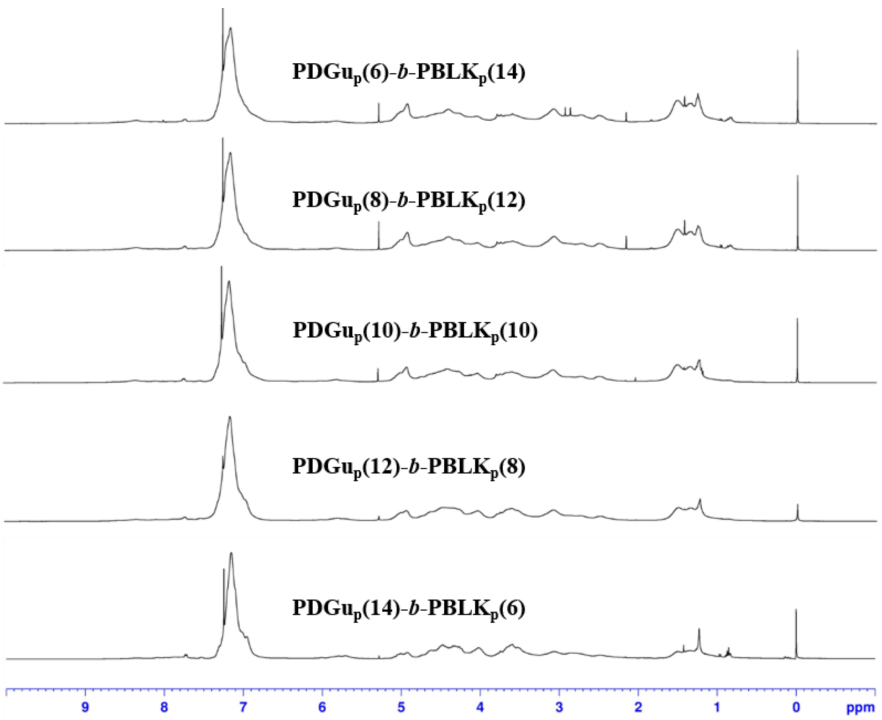

Supplementary Figure 6 ^{1}H NMR spectra of **PDGu$_p$(x)-*b*-PBLK$_p$(y)** in $CDCl_3$.

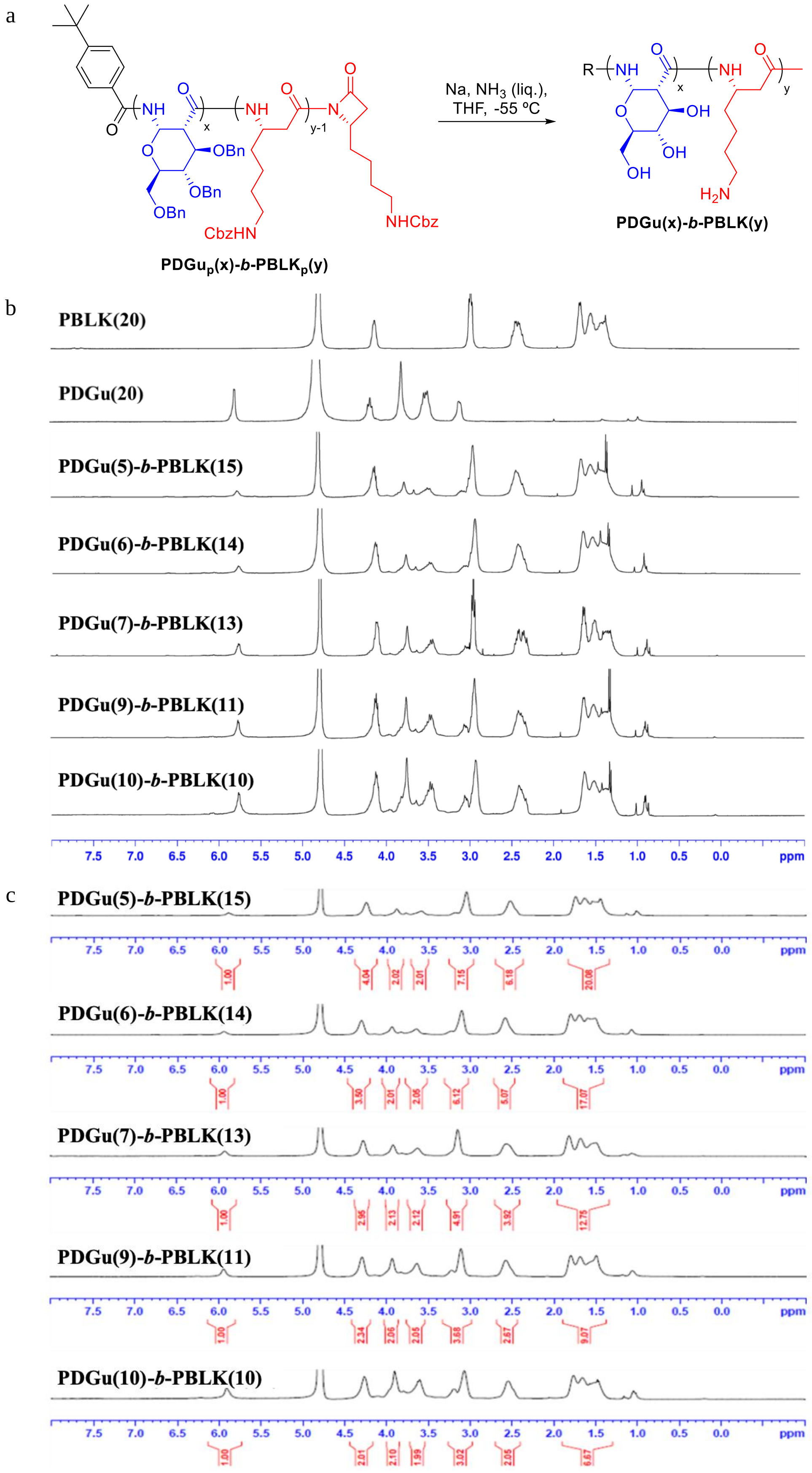

Supplementary Figure 7 Deprotection and characterization of **PDGu(x)-b-PBLK(y)**. **(a)** Deprotection scheme **(b)** rt & **(c)** 50 °C ^{1}H NMR spectra in D_2O.

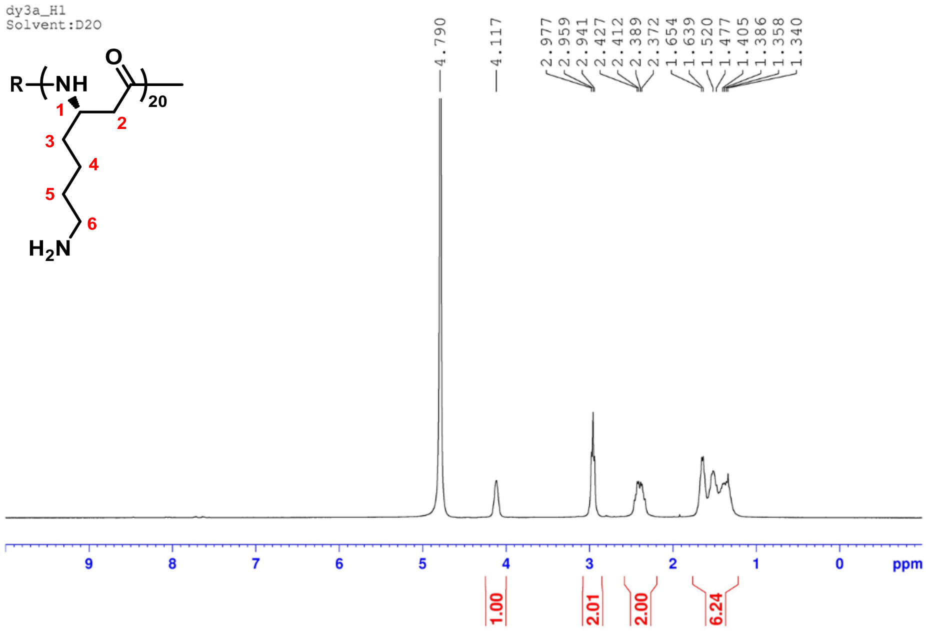

Supplementary Figure 8 ^{1}H NMR spectra of **PBLK(20)**.

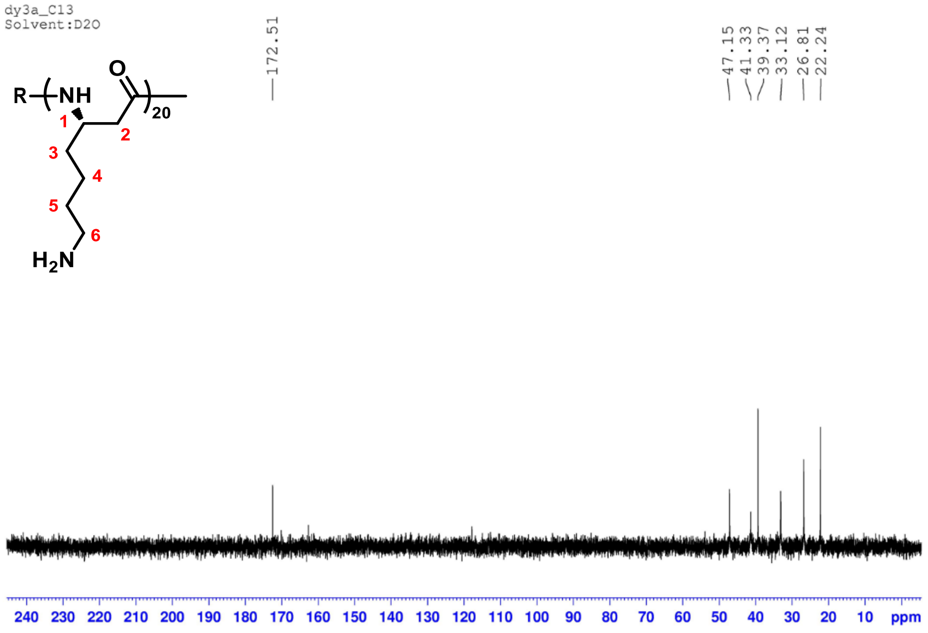

Supplementary Figure 9 ^{13}C NMR spectra of **PBLK(20)**.

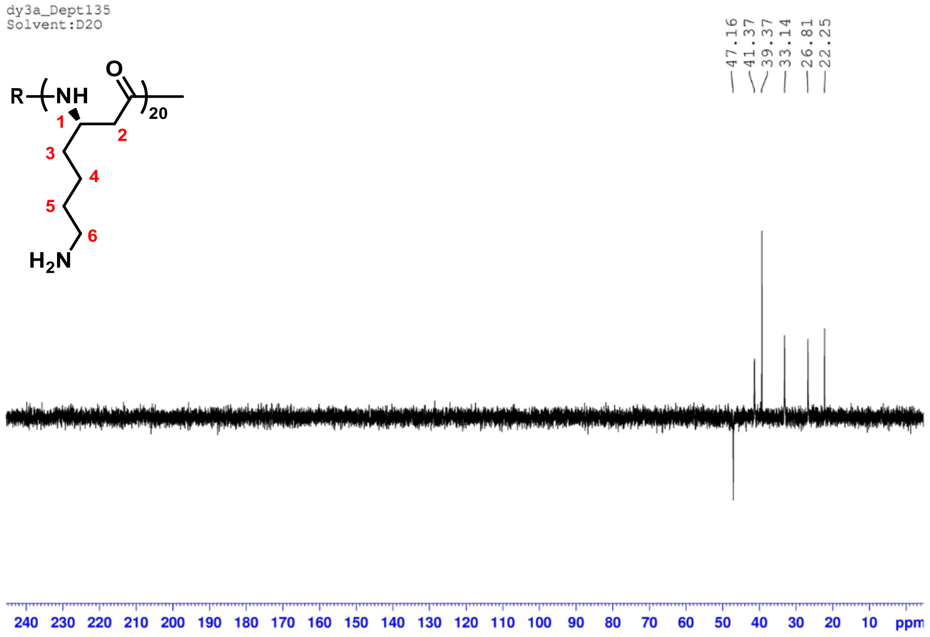

Supplementary Figure 10 DEPT135 NMR spectra of **PBLK(20)**.

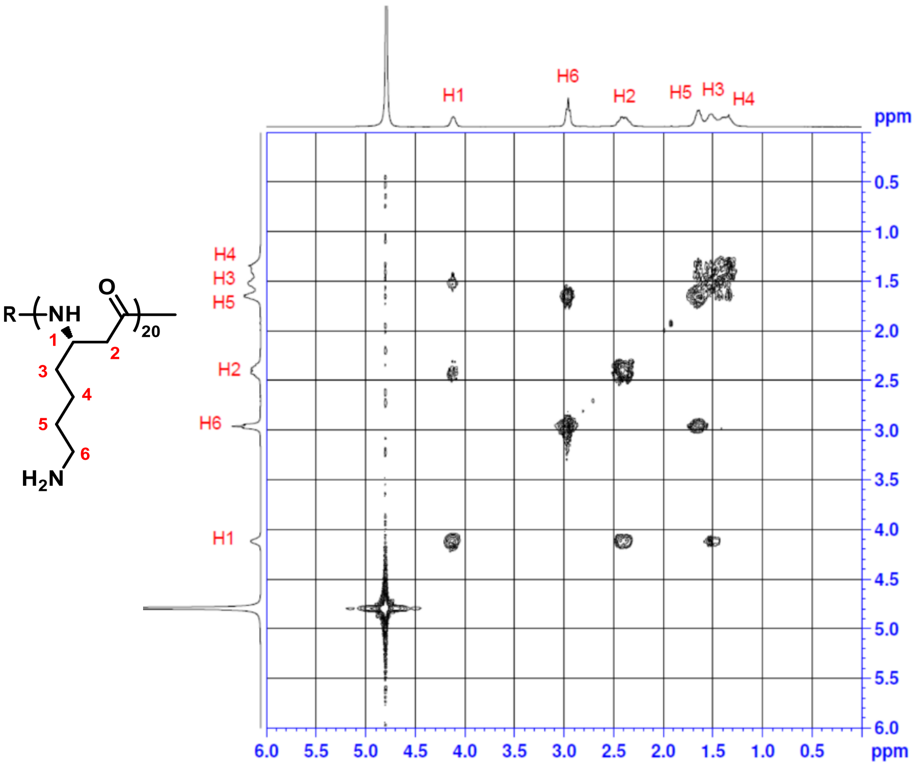

Supplementary Figure 11 COSY NMR spectra of **PBLK(20)**.

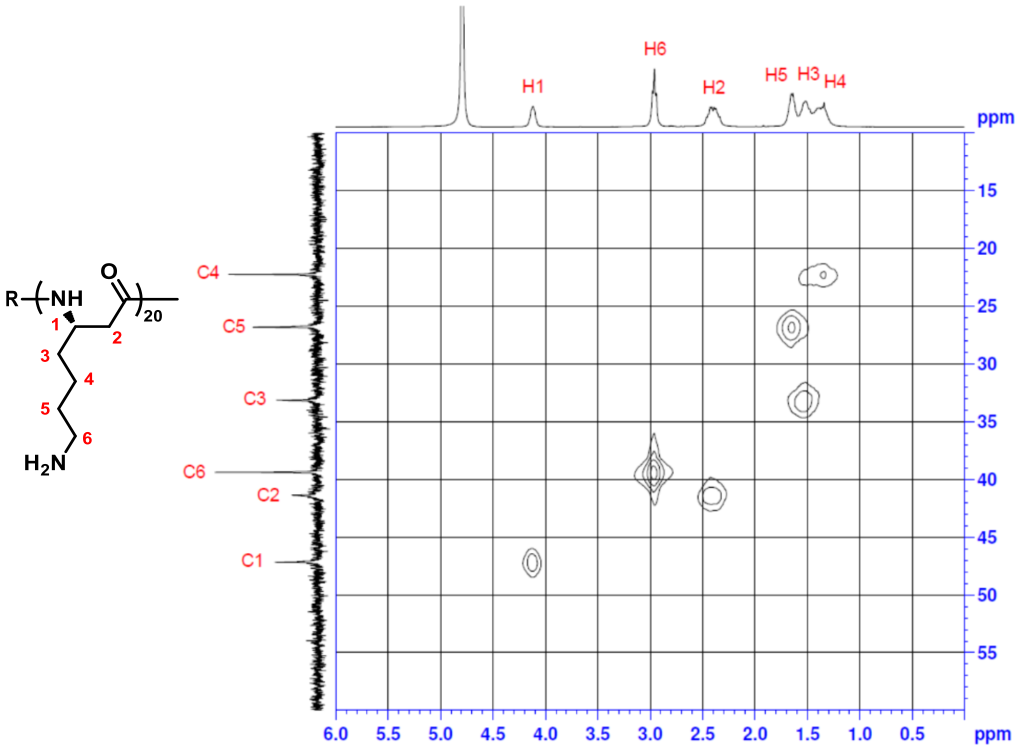

Supplementary Figure 12 HMQC NMR spectra of **PBLK(20)**.

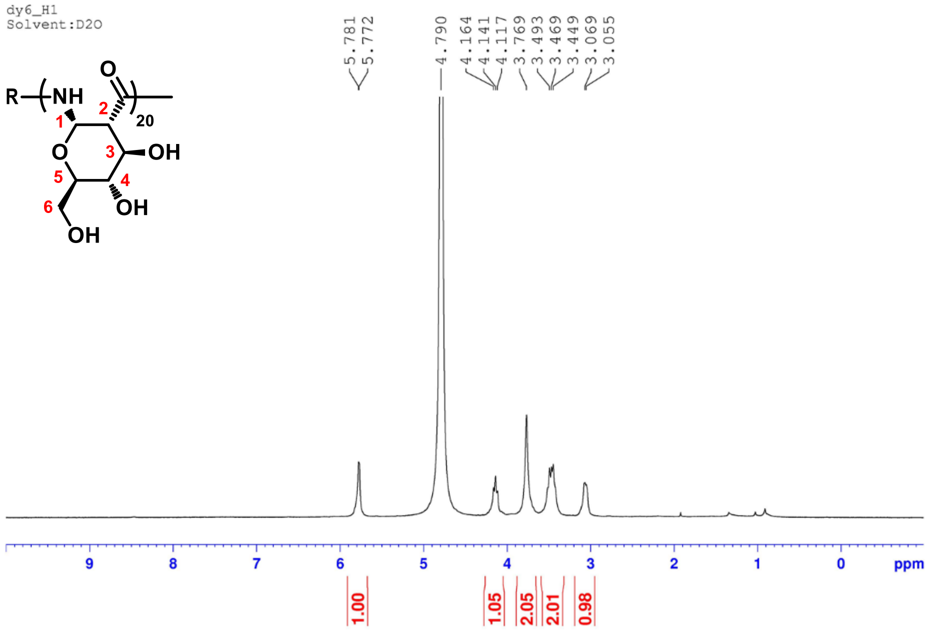

Supplementary Figure 13 ^{1}H NMR spectra of **PDGu(20)**.

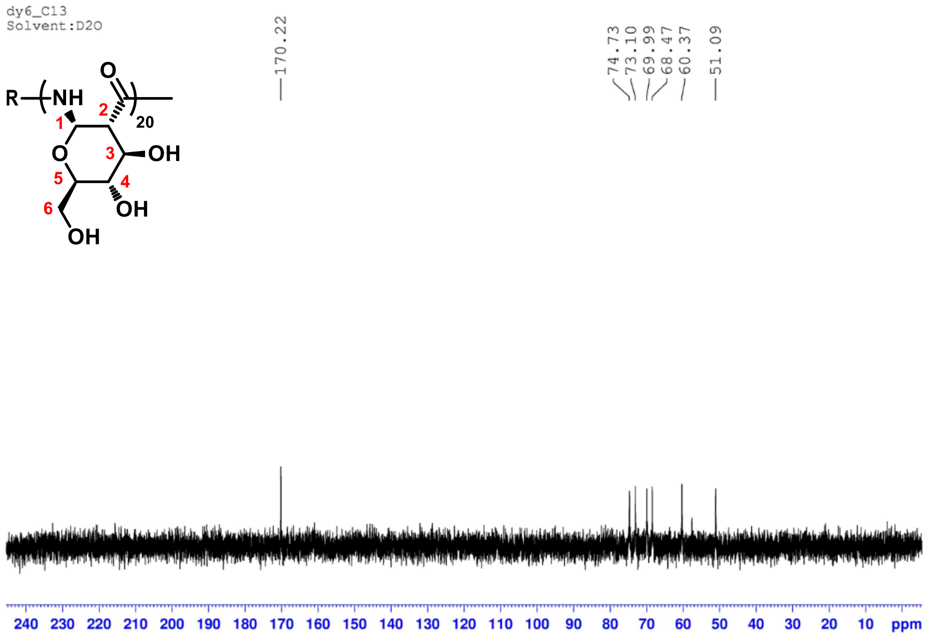

Supplementary Figure 14 ^{13}C NMR spectra of **PDGu(20)**.

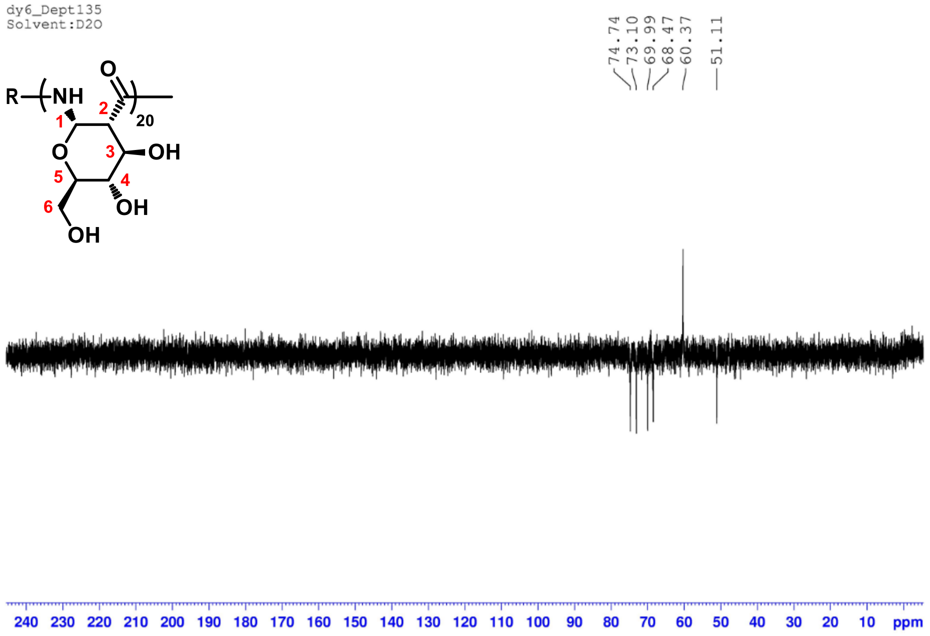

Supplementary Figure 15 DEPT135 NMR spectra of **PDGu(20)**.

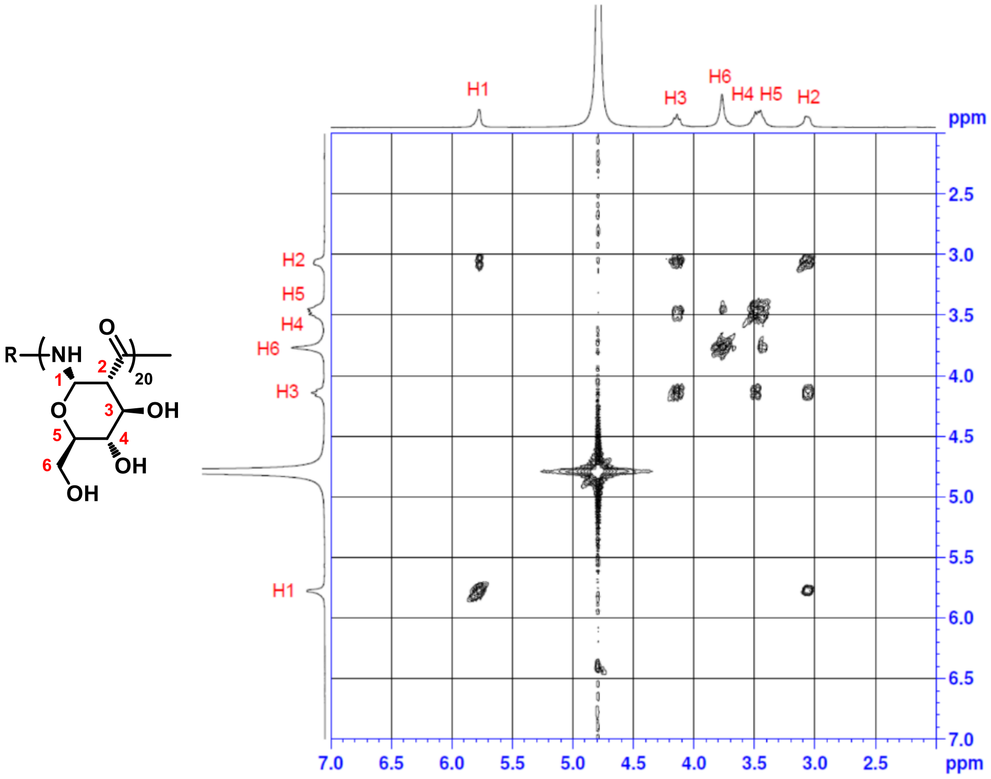

Supplementary Figure 16 COSY NMR spectra of **PDGu(20)**.

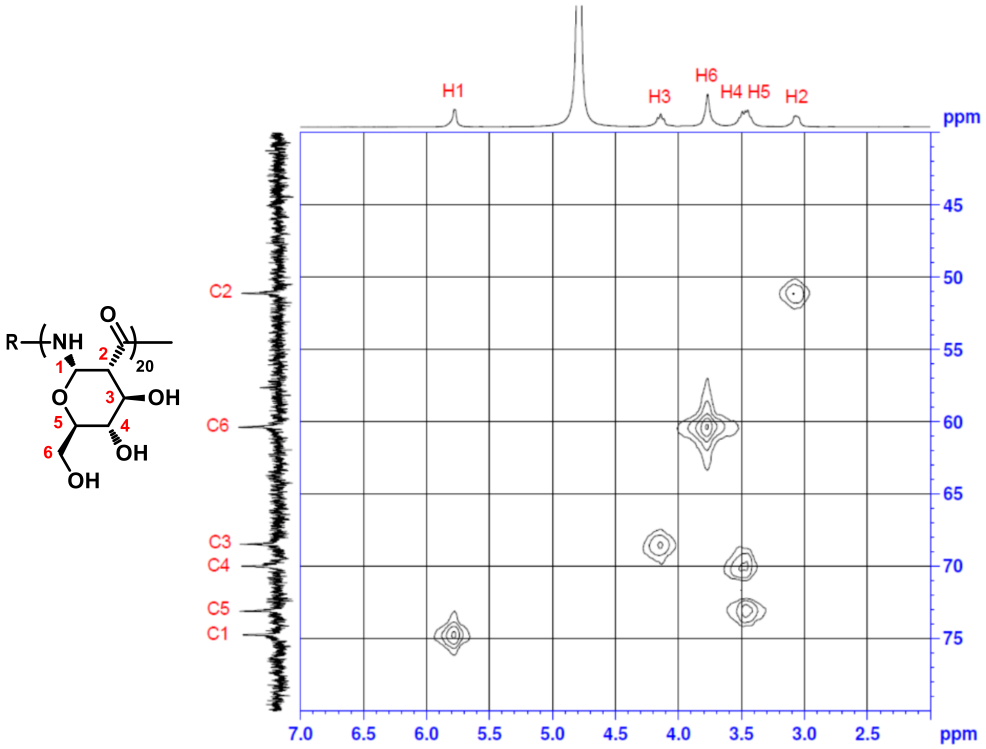

Supplementary Figure 17 HMQC NMR spectra of **PDGu(20)**.

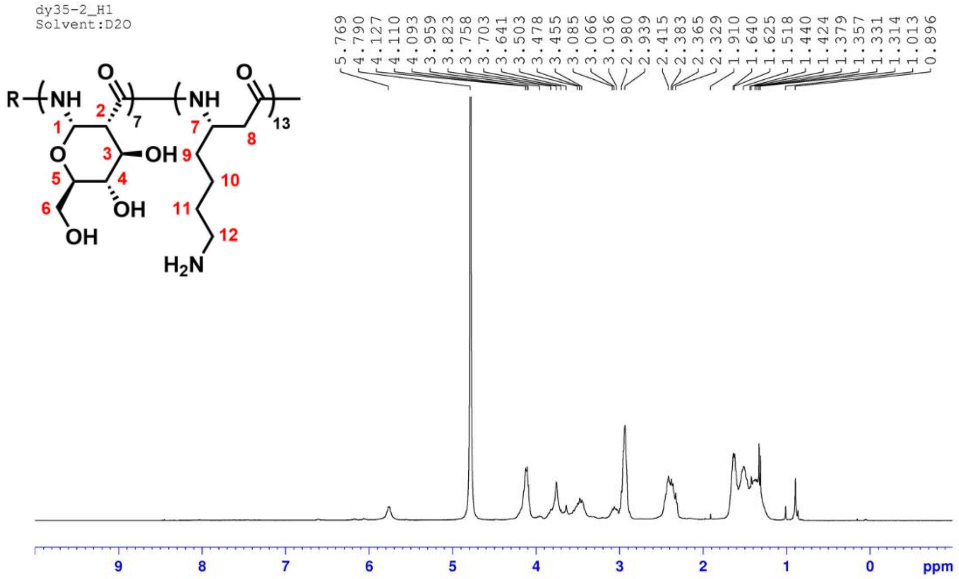

Supplementary Figure 18 ^{1}H NMR spectra of **PDGu(7)-*b*-PBLK(13)**.

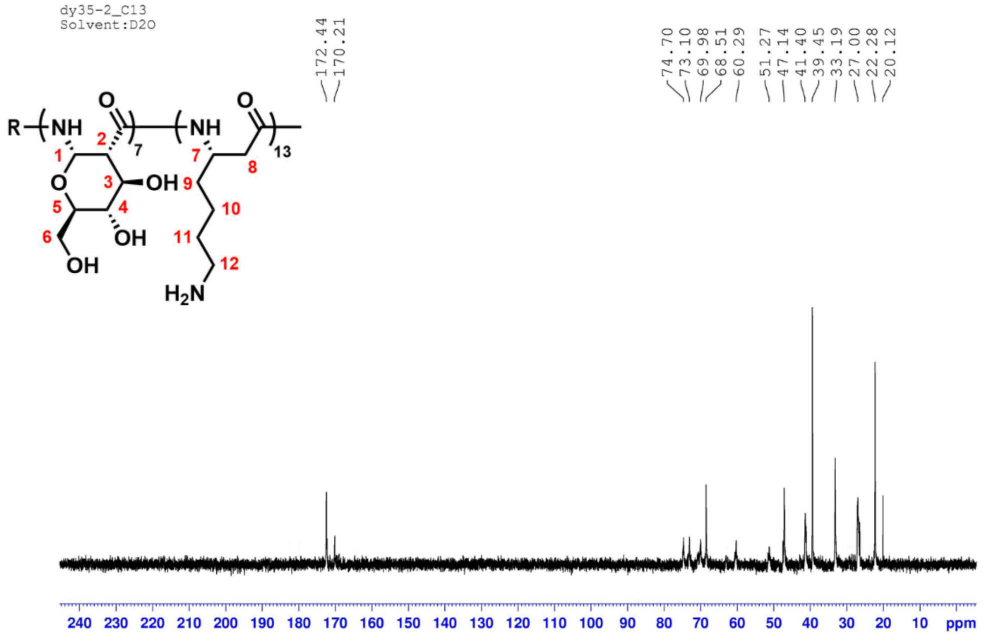

Supplementary Figure 19 ^{13}C NMR spectra of **PDGu(7)-*b*-PBLK(13)**.

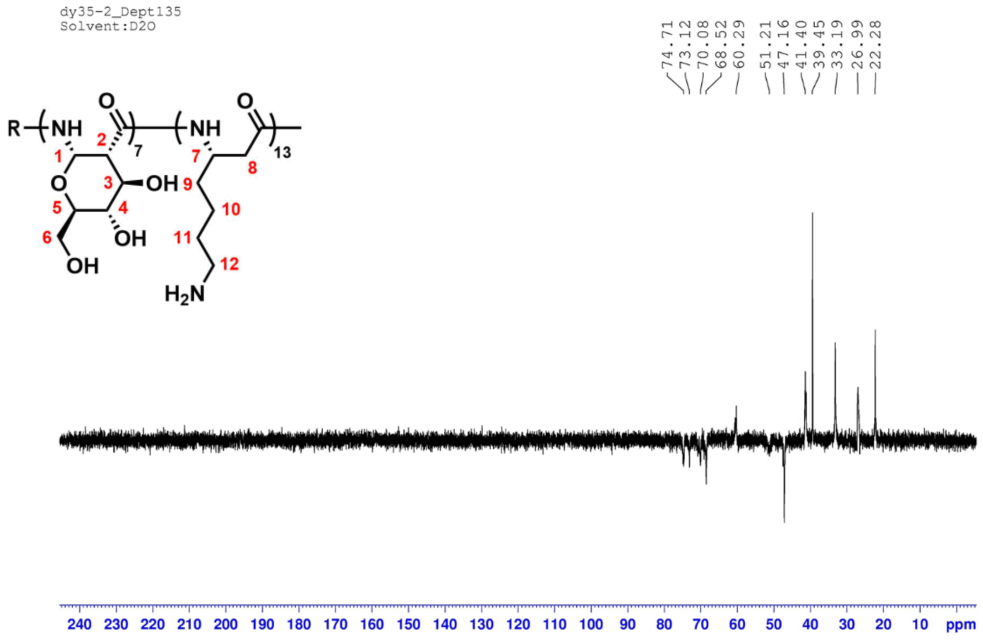

Supplementary Figure 20 DEPT135 NMR spectra of **PDGu(7)-*b*-PBLK(13)**.

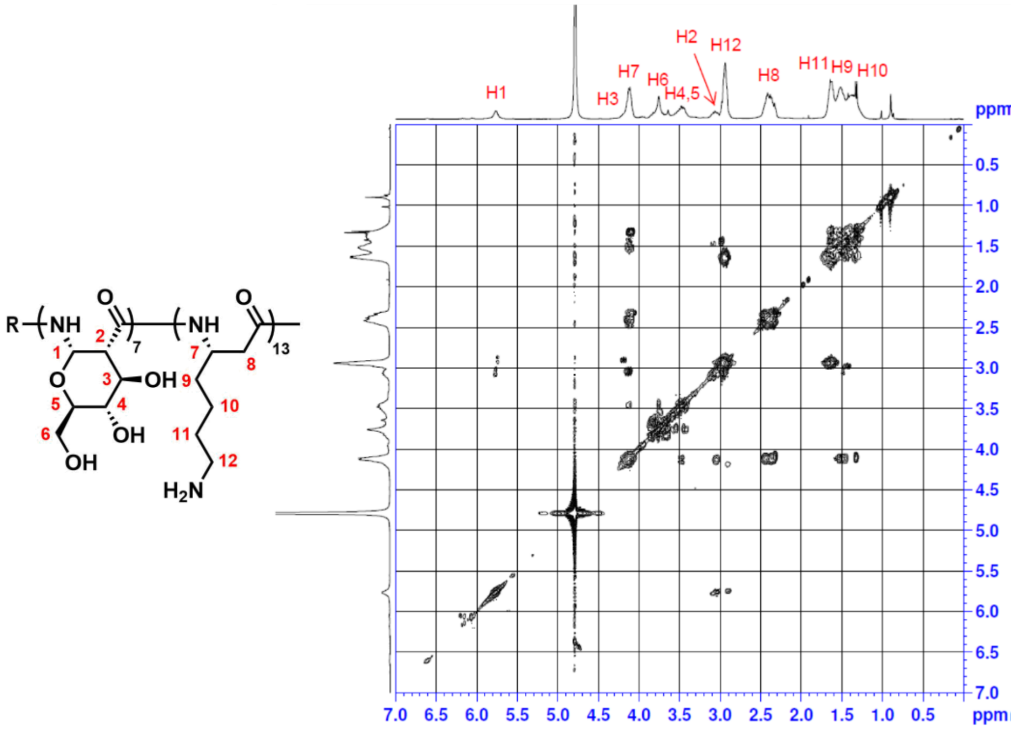

Supplementary Figure 21 COSY NMR spectra of **PDGu(7)-*b*-PBLK(13)**.

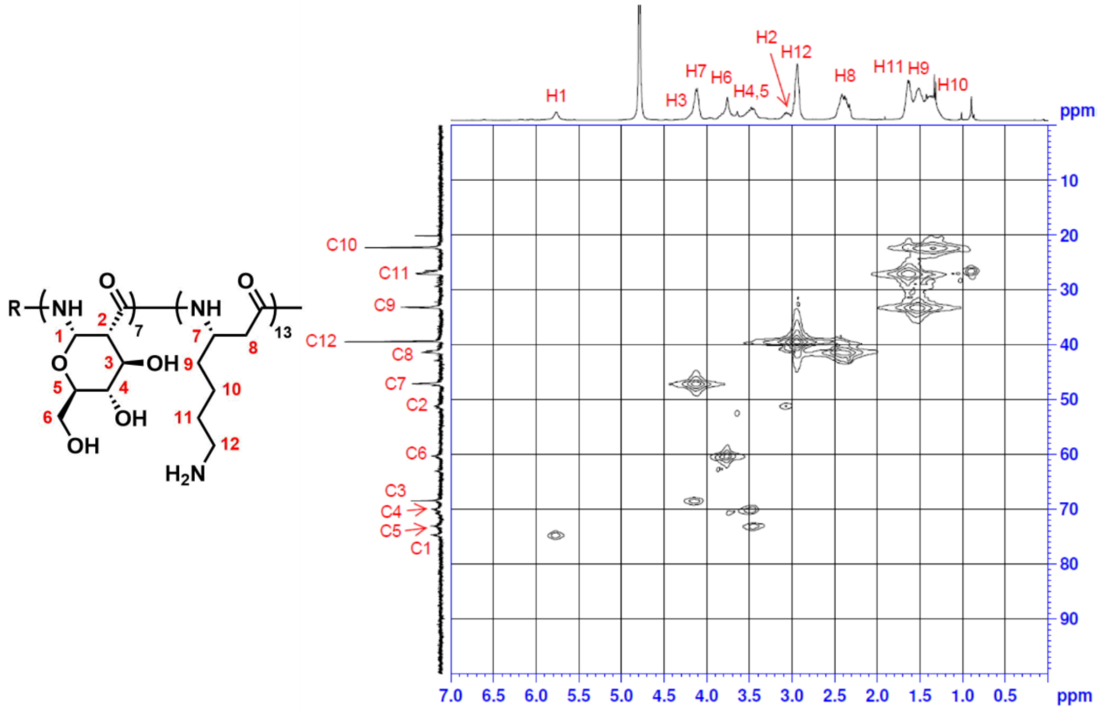

Supplementary Figure 22 HMQC NMR spectra of **PDGu(7)-*b*-PBLK(13)**.

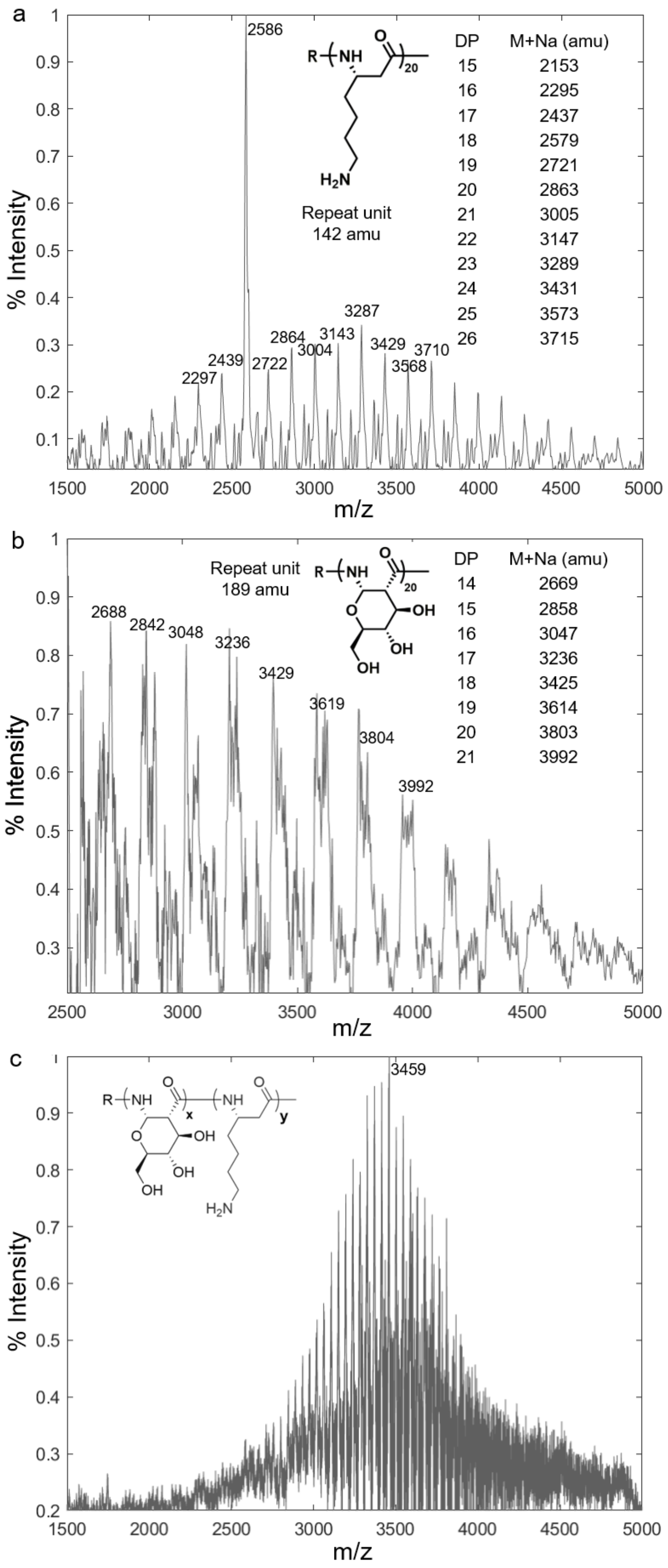

Supplementary Figure 23 MALDI-TOF analysis. **(a)** homocationic **PBLK(20)**, **(b)** homosugar **PDGu(20)** and **(c)** block copolymer **PDGu(7)-*b*-PBLK(13)**.

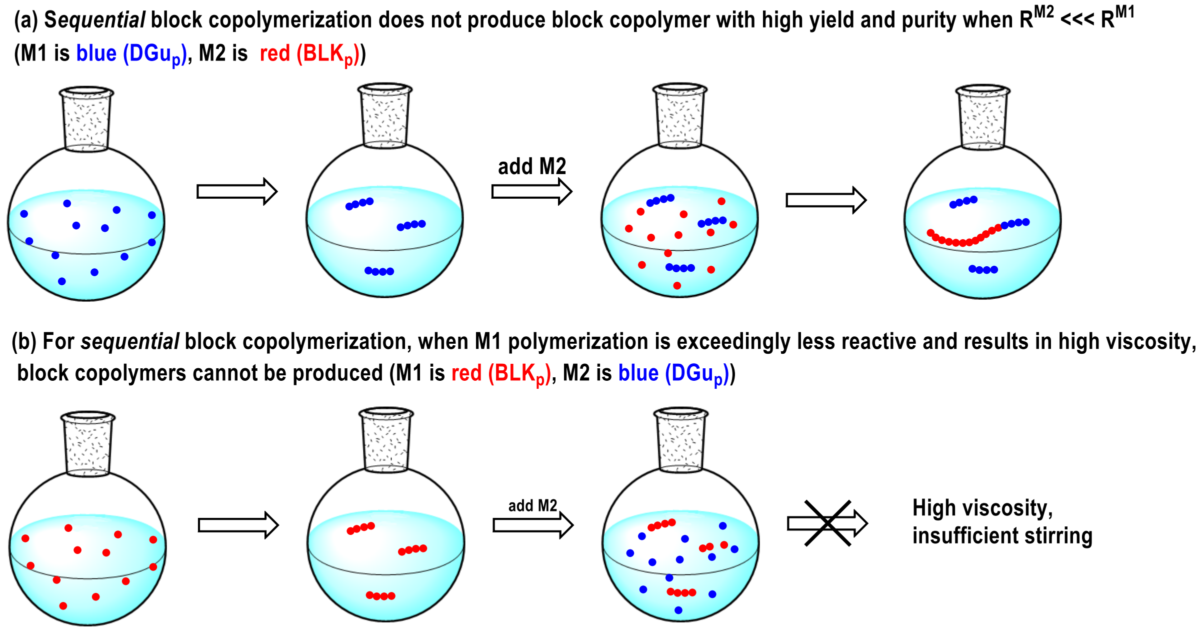

Supplementary Figure 24 *Sequential* block copolymerization approach does not produce block copolymer well for the pair of **BLK**$_\mathbf{p}$ and **DGu**$_\mathbf{p}$ regardless of the sequence of monomer addition. **(a)** Addition of **DGu**$_\mathbf{p}$ first. **(b)** Addition of **BLK**$_\mathbf{p}$ first.

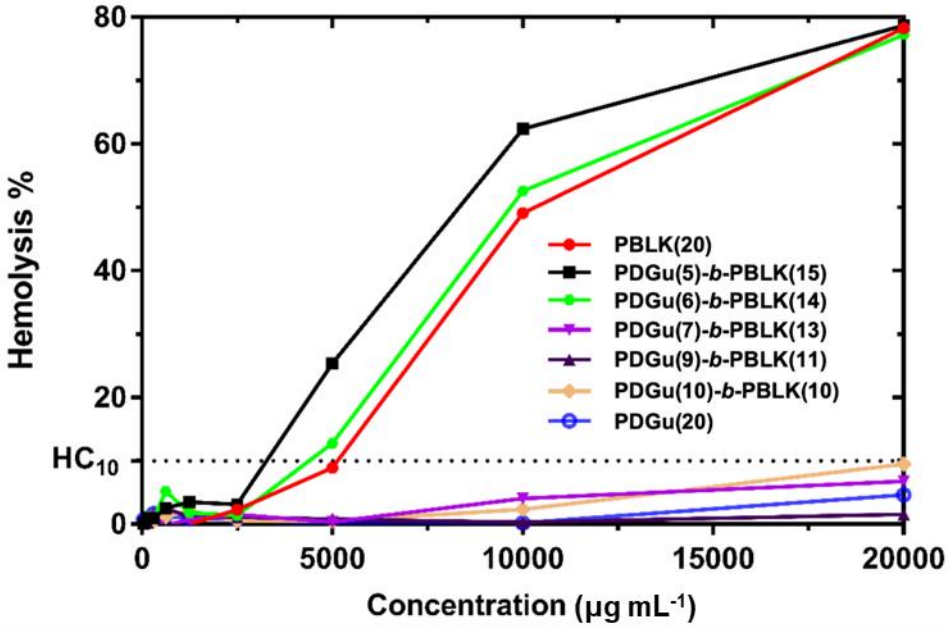

Supplementary Figure 25 Hemocompatibility of **PDGu(x)-*b*-PBLK(y)**

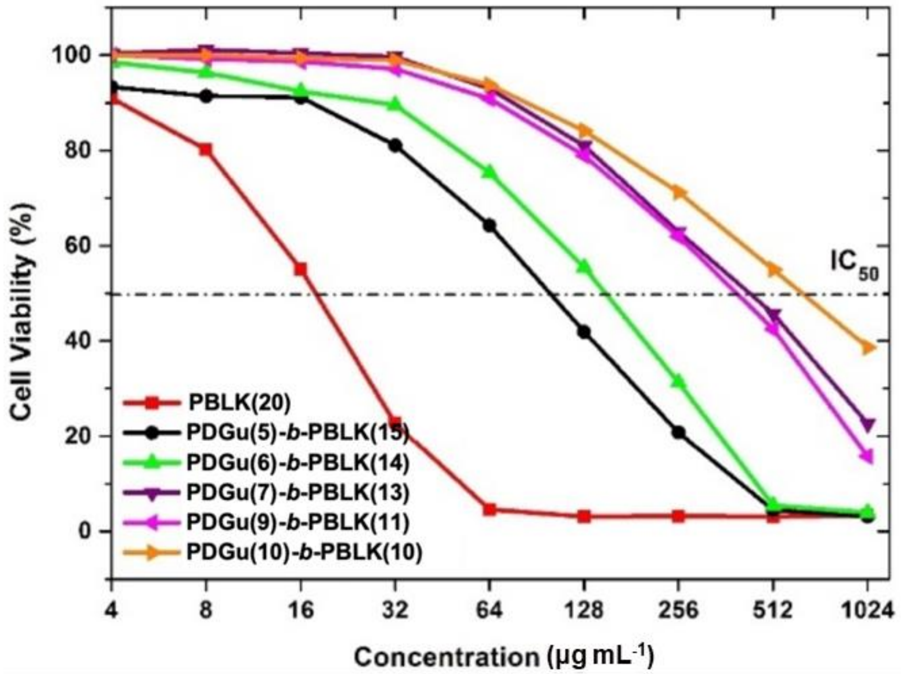

Supplementary Figure 26 In vitro cytotoxicity assay. **PDGu(x)-*b*-PBLK(y)** series 24-hour acute toxicity against 3T3 mouse fibroblast cells.

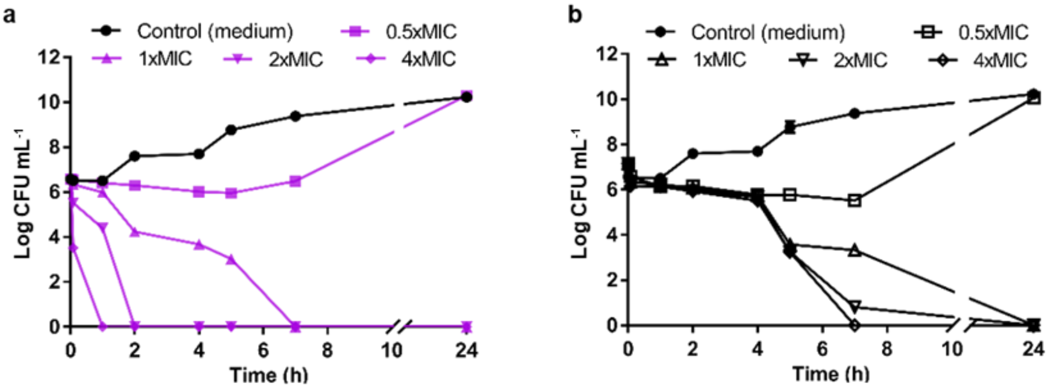

Supplementary Figure 27 Time-dependent killing of actively growing MRSA USA300 at 4×MIC, 2×MIC, 1×MIC and 0.5×MIC. **(a) PDGu(7)-*b*-PBLK(13)** and **(b)** vancomycin in MHB.

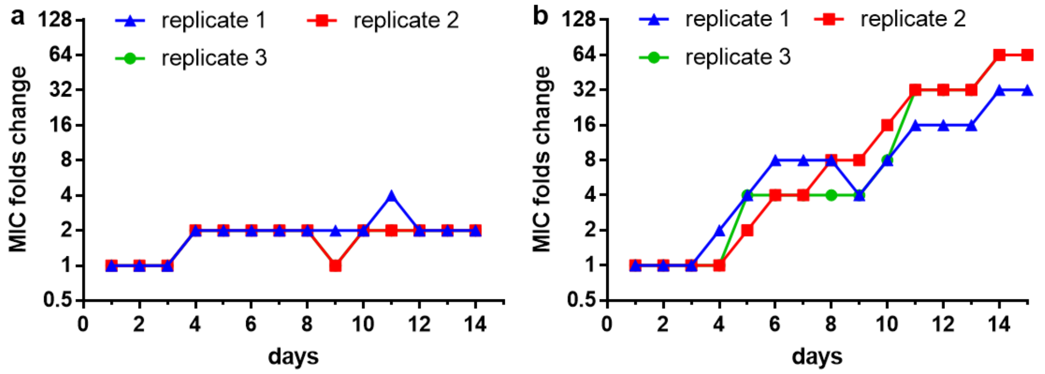

Supplementary Figure 28 Resistance development of MRSA USA300 by serial passage with sub-inhibitory concentrations of the agent. Data are presented as folds MIC change over time. **(a) PDGu(7)-*b*-PBLK(13)** and **(b)** Ciprofloxacin antibiotic control. All tests were done with three biological replicates.

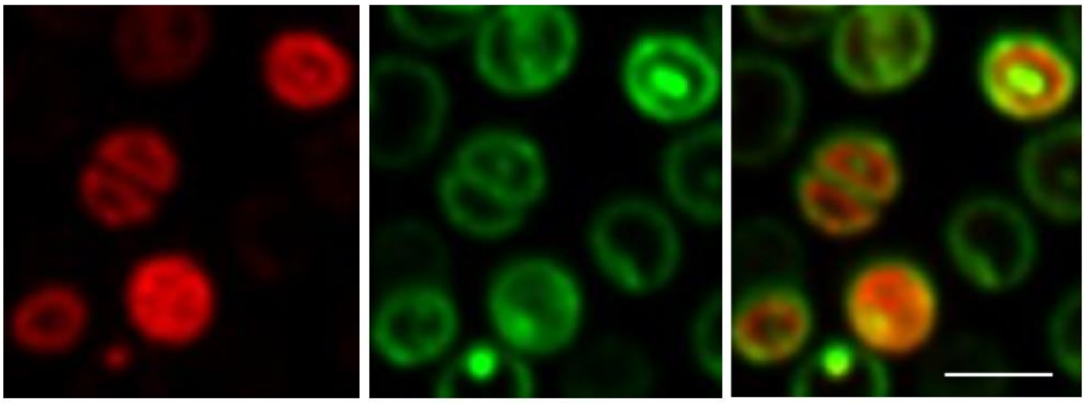

Supplementary Figure 29 Confocal image of homocationic **PBLK(20)** treated MRSA USA300 (10^8 CFU mL^{-1} in PBS, 16 μg mL^{-1} for 1 hour). Scale bar = 1 μm. From left to right: Rhodamine-labelled homopolymer channel, FM1-43 labelled bacteria membrane channel, superimposed images from both channels, respectively.

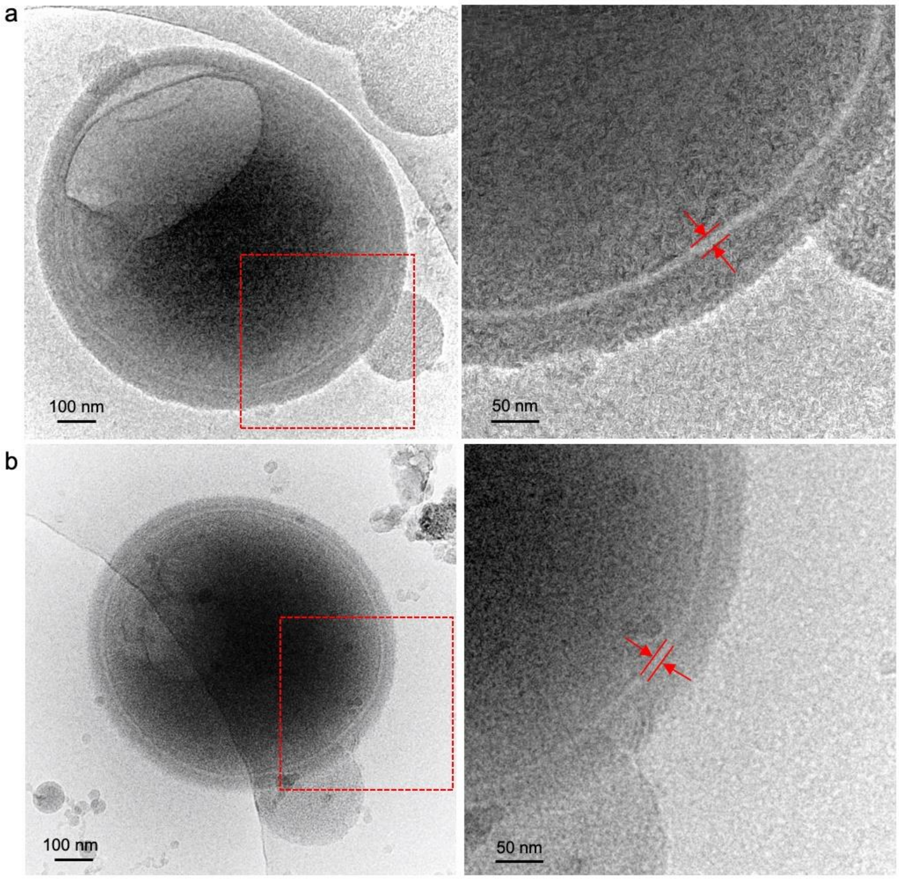

Supplementary Figure 30 Cryo-TEM images of **PDGu(7)-*b*-PBLK(13)** treated MRSA USA300 (10^8 CFU mL^{-1} in PBS). This figure consists of two representative sets of images (a and b). The right panels are zoom-in images of the left panels.

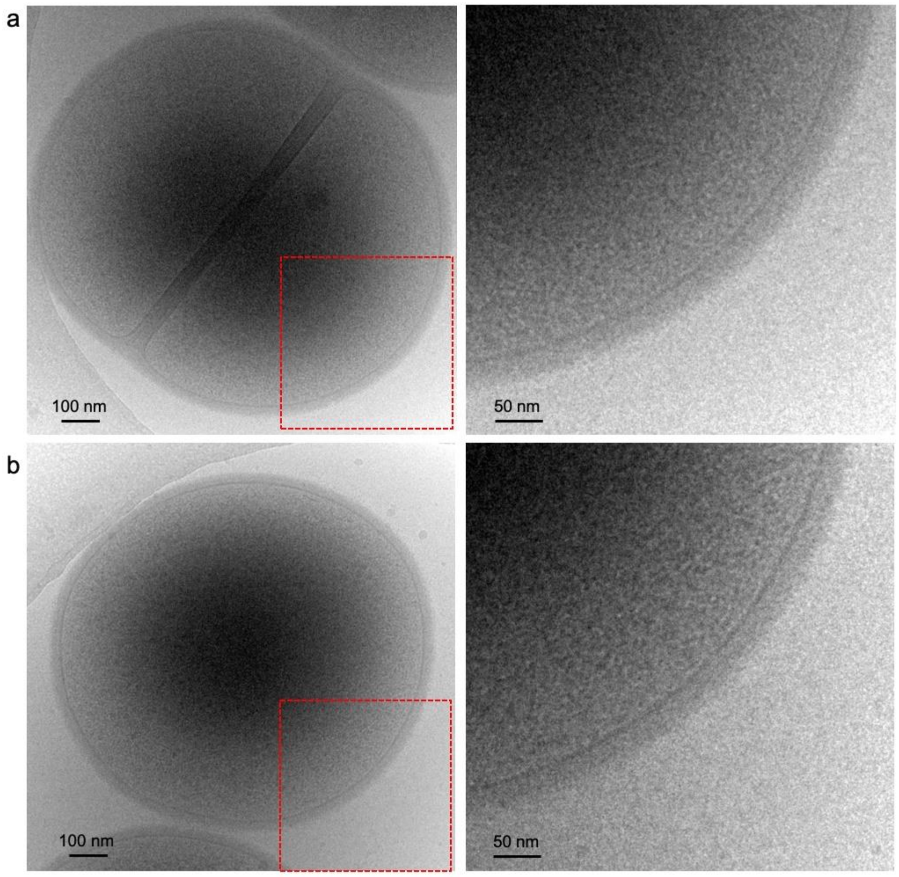

Supplementary Figure 31 Cryo-TEM images of untreated MRSA USA300 control (10^8 CFU mL^{-1} in PBS). This figure consists of two representative sets of images (a and b). The right panels are zoom-in images of the left panels.

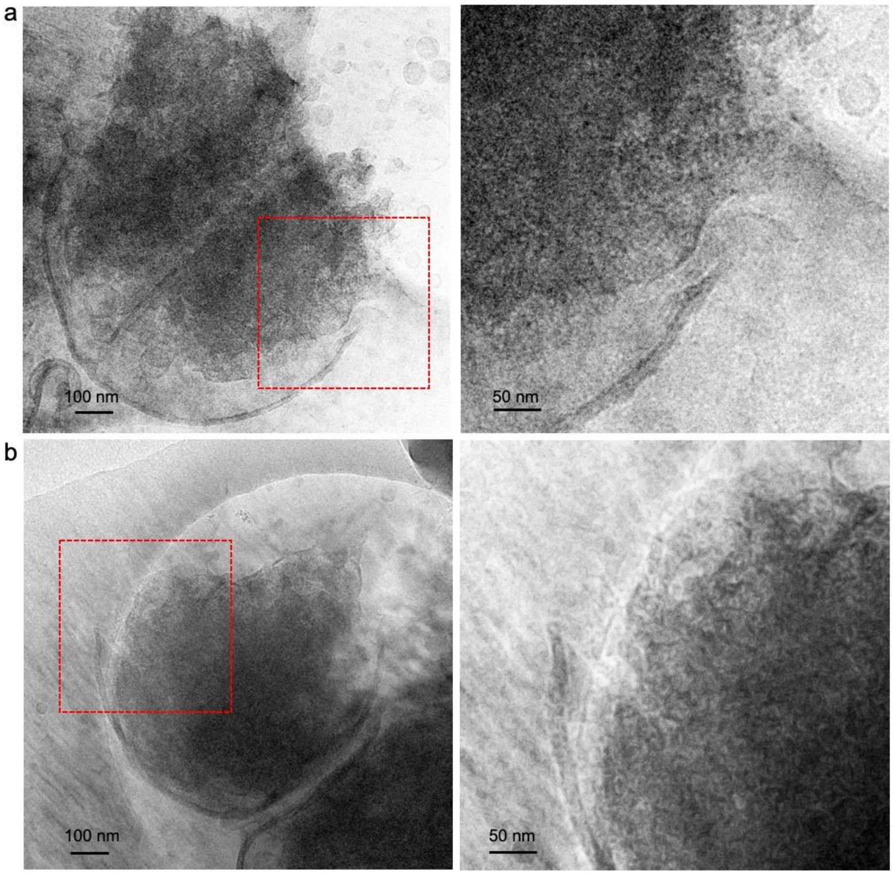

Supplementary Figure 32 Cryo-TEM images of **PBLK(20)** treated MRSA USA300 (10^8 CFU mL^{-1} in PBS). This figure consists of two representative sets of images (a and b). The right panels are zoom-in images of the left panels.

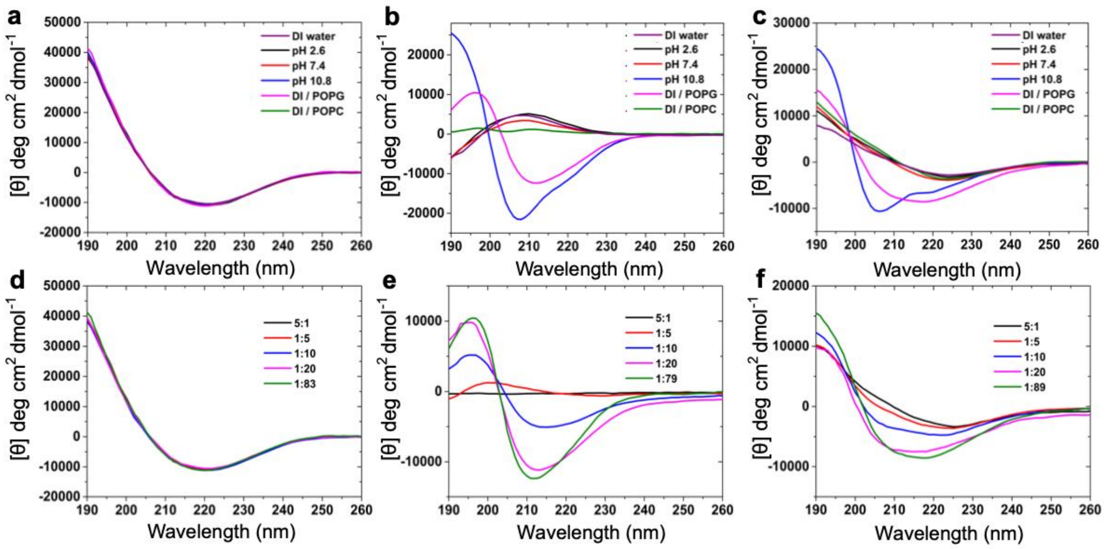

Supplementary Figure 33 Secondary structure study using circular dichroism. **(a to c)** Molar ellipticity [θ] circular dichroism spectra in different media *i.e.* DI water, 10 mM phosphate buffer (pH 2.6-8.7), 20 mM carbonate buffer (pH 10.8) and in the presence of POPG liposomes (model bacterial membrane) and POPC liposomes (model mammalian membrane). (a) **PDGu(20)**, (b) **PBLK(20)** and (c) **PDGu(7)-*b*-PBLK(13)** at 0.05 mg mL^{-1}. **(d to f)** Molar ellipticity [θ] circular dichroism spectra in the presence of anionic POPG liposome at different P:L ratios. (d) **PDGu(20)**, (e) **PBLK(20)** and (f) **PDGu(7)-*b*-PBLK(13)**.

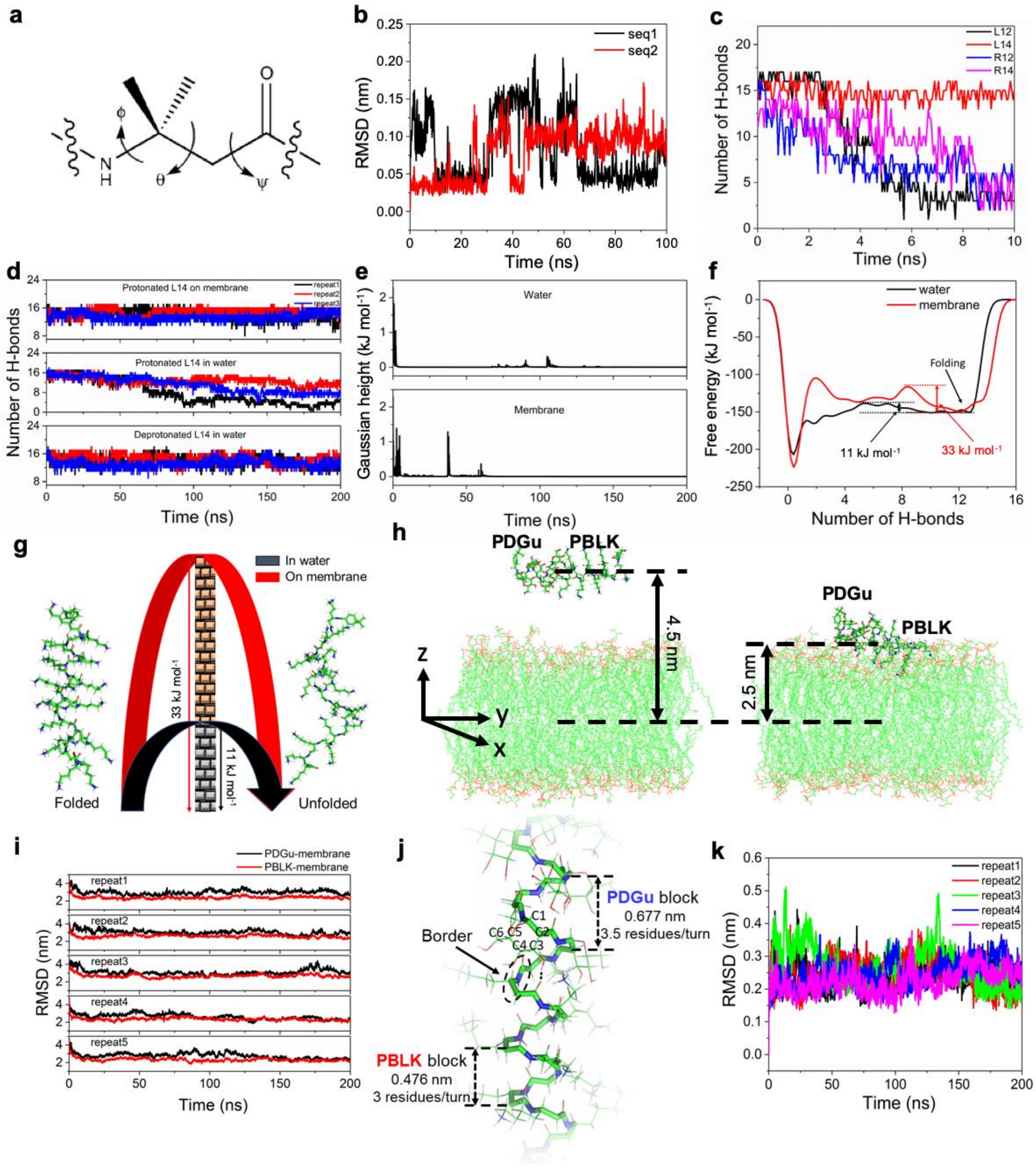

Supplementary Figure 34 Secondary structure study using computer simulation. **(a)** Definition of the three backbone dihedral angles in a typical beta-peptide. **(b)** Two additional benchmark simulations to confirm the validity of the force field parameters for beta-peptide. **(c)** Time evolution of the intramolecular H-bonds of different PBLK helices under the membrane environment. Only the L14 helix is stable. **(d)** Time evolution of the intramolecular H-bonds of the L14 helix. The electrostatic repulsion among the PBLK side chains destabilize the helix (Protonated L14 in water). Neutralization or removal of the charges stabilize the helical structure (Protonated L14 on membrane and Deprotonated L14 in water). **(e)** Gaussian height decays to a small value indicating the convergence of the well-tempered meta-dynamics. **(f)** The folding free energy profiles of **PBLK(20)** in different environments. **(g)** The transition from the

folded state to the unfolded state in the membrane environment is more difficult than in water environment because of the higher free energy barrier. **(h)** Left panel: The initial configuration of the beta-peptide & membrane system. Right panel: The bound configuration of the beta-peptide & membrane system. The carbon, hydrogen, oxygen, nitrogen and phosphorus atoms are colored as green, white, red, blue, and orange, respectively. The same color scheme is used for the rest structure figures. **(i)** The Z-Distance between each of the blocks of the copolymer and the membrane. **(j)** H-bond formation in the border between the **PDGu** and the **PBLK** blocks of the **PDGu(7)-*b*-PBLK(13)**. The height and number of residues per helical turn are different in the two helices. The copolymer backbone is shown as stick model while the rest of molecule is shown as line model. **(k)** RMSD value of the **PDGu(7)-*b*-PBLK(13)** heavy atoms with respect to those of the initial structure.

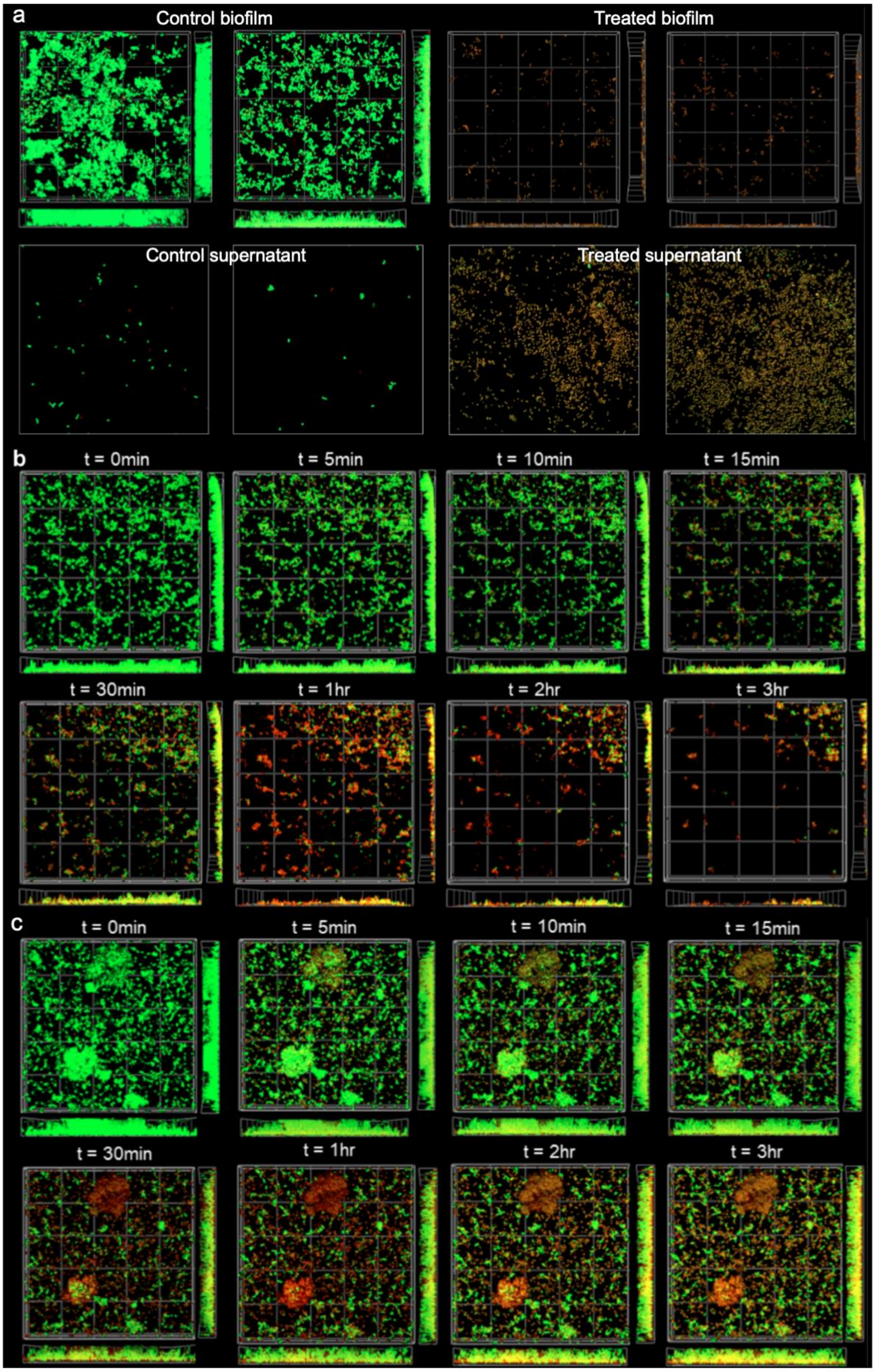

Supplementary Figure 35 Confocal image of biofilm treated with polymers. **(a)** Confocal image of biofilm bacteria on collagen-coated glass bottom petri dish and dispersed bacteria in supernatant after copolymer treatment. Bacteria stained with Live/Dead *Bac*Light™ dye. Upper panel: biofilm bacteria on petri dish. Lower panel:

supernatant bacteria on top of biofilm. (left) PBS control with minimal bacteria dispersed from biofilm; both biofilm and supernatant bacteria remain alive; (right) Dispersed biofilm after treatment with 32 μg mL^{-1} **PDGu(7)-*b*-PBLK(13)** in PBS for 3 hours under static condition. Biofilm on petri dish was dispersed and majority of bacteria released from biofilm are dead, indicated by PI staining. **(b)** Time lapse confocal images of biofilm stained with Live/Dead *Bac*Light™ dye. Treated with 32μg mL^{-1} **PDGu(7)-*b*-PBLK(13)**. **(c)** Time lapse confocal images of biofilm stained with Live/Dead BacLight dye. Treated with 32μg mL^{-1} **PBLK(20)**. Z stack thickness = 10 μm.

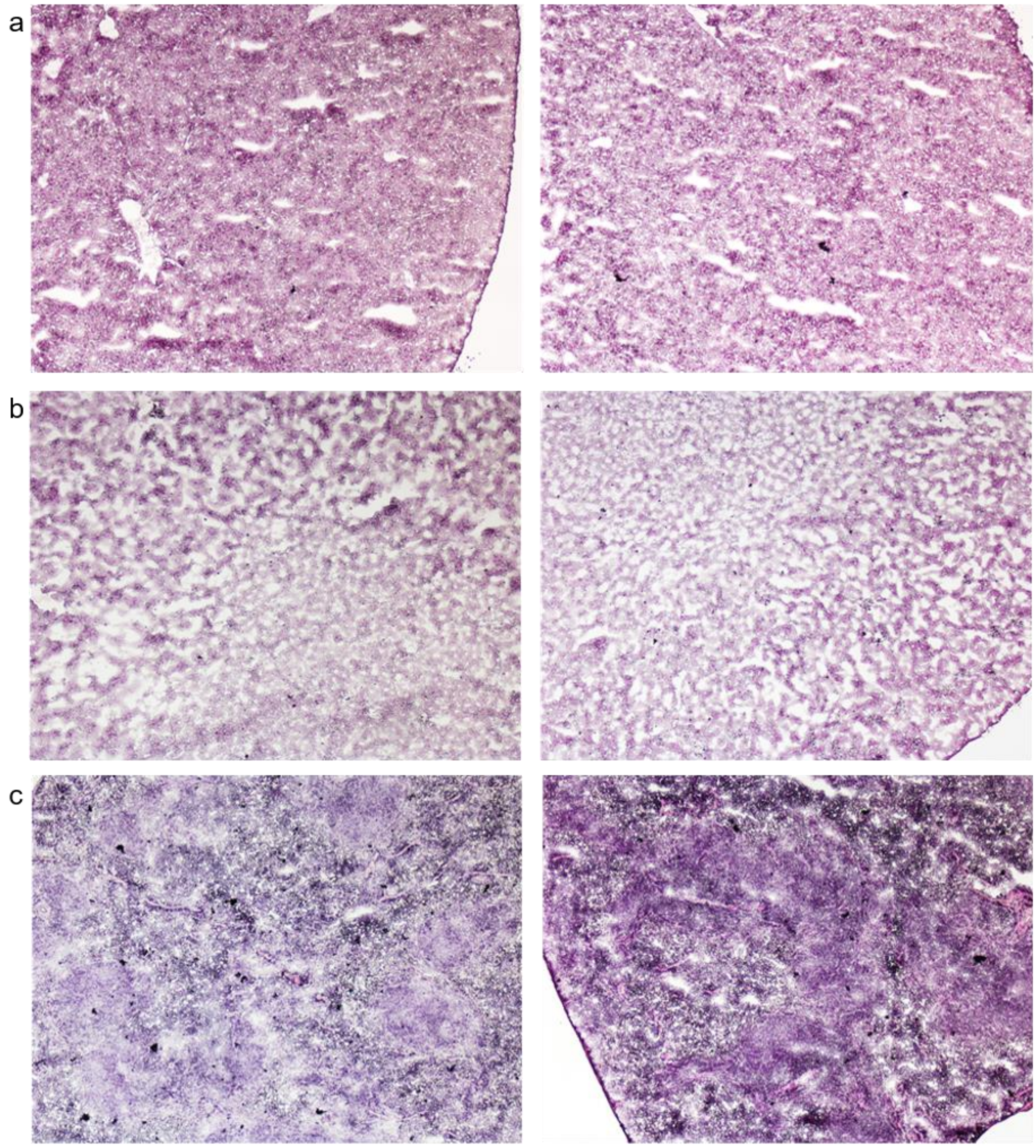

Supplementary Figure 36 In vivo toxicity test in murine intravenous injection model. **PDGu(7)-*b*-PBLK(13)** injected at 10.0 mg kg^{-1} daily for 7 days. **(a to c)** Histology of organs of untreated (left) and copolymer treated (right) mice. **(a)** Kidney **(b)** Liver **(c)** Spleen.

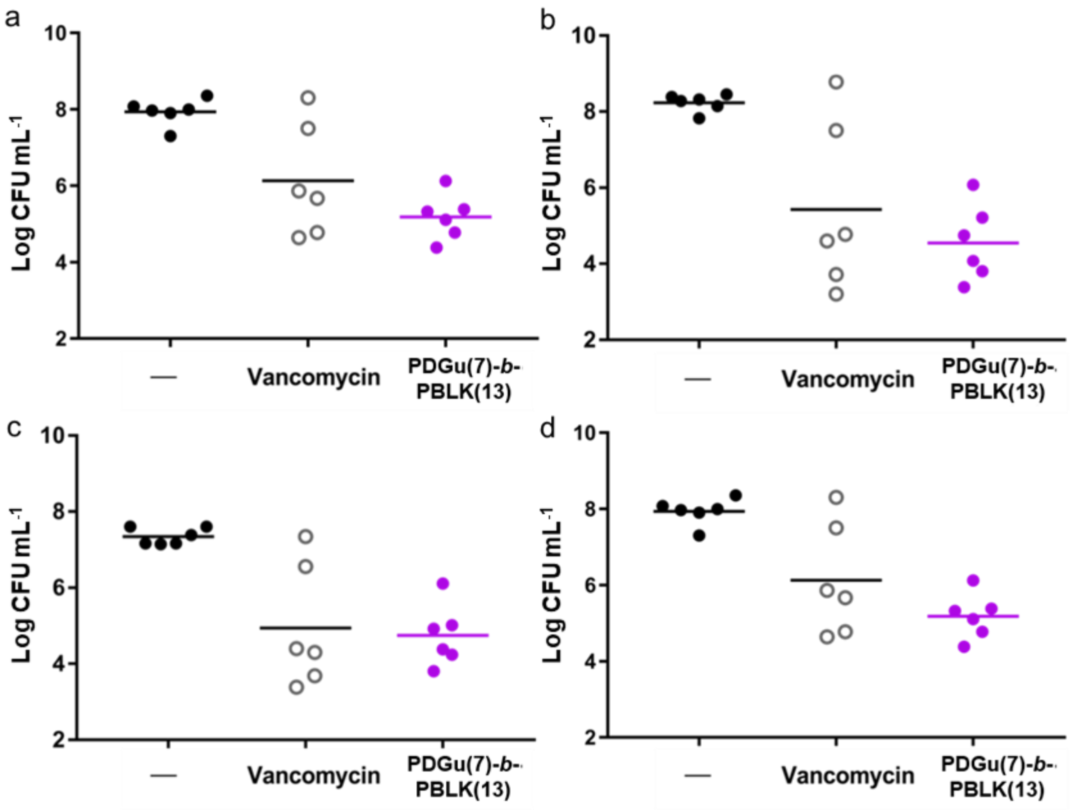

Supplementary Figure 37 In vivo efficacy test in murine sepsis model**.** Vehicle alone (–), vancomycin and **PDGu(7)-*b*-PBLK(13)** at 5 mg kg^{-1} were injected i.p. and bacteria loads were expressed as log CFU of **(a)** liver, **(b)** IP fluid, **(c)** kidney and **(d)** spleen**.**

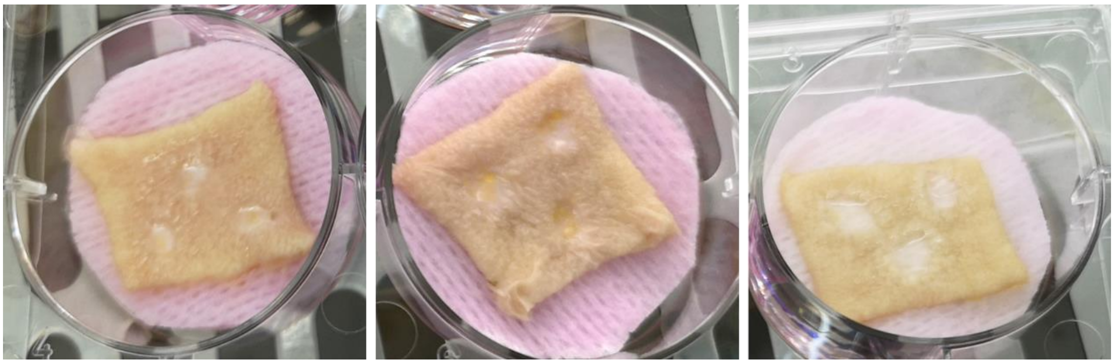

Supplementary Figure 38 A representative set of wound images by end of the treatment. From left to right: Vehicle alone, vancomycin and copolymer treated samples.

Supplementary Tables

Supplementary Table 1. Screening of monomers via relative rates of homopolymerization[a]

Monomer	Reaction time for homopolymerization[a]
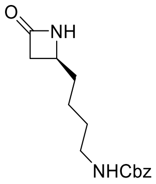 *N*-Cbz-*β*-lactam-L-hLys (**BLK$_p$**)	8 hours[b]
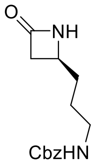 *N*-Cbz-*β*-lactam-L-hOrnithine	6 hours
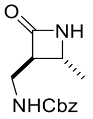 *N*-Cbz-MM*β*4	2 hours
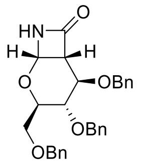 *O*-Bn-*β*-lactam-D-glucose (**DGu$_p$**) 5	10 min
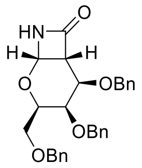 *O*-Bn-*β*-lactam-D-galactose 6	10 min

[a] Approximate reaction time for complete homopolymerization performed at −30 ºC. Theoretical degree of polymerization (DP_{theo}) = 20. [b] The well-controlled AROP synthesis of poly-beta-L-hLys (**PBLK**) has not been reported previously. Room temperature polymerization leads to the reaction finishing immediately after initiation by base, which also yielded the protected polymer **PBLK$_p$** with a higher molecular weight than anticipated based on stoichiometry. The initiation rate of **BLK$_p$** appears to be much slower than its rate of propagation, which is undesirable. Reducing the reaction temperature reduces both initiation and propagation rates. When AROP was carried out at −30 ºC, the degree of polymerization of **PBLK$_p$(20)** (DP_{theo} = 20) matched the stoichiometric value. Complete homopolymerization of the monomer **BLK$_p$** requires about 8 hours at −30 ºC, while the monomer is essentially quantitatively recovered if reacted for only 10 minutes. After deprotection by sodium in ammonia, the molecular weight of the water-soluble **PBLK(20)** calculated based by using gel permeation chromatography (GPC) was close to the theoretical value with an acceptable dispersity (*Đ* = 1.2).

Supplementary Table 2. Molecular weights and polydispersities of (co)polymers, and ratios of **DGu_p** to **BLK_p** in **$PDGu_p(x)$-*b*-$PBLK_p(y)$**

Sample	Design ratio of DGu_p to BLK_p	Calculated ratio[a] of DGu_p to BLK_p	$M_{n,theo}$ (Da)	$M_{n, GPC}$[a] (Da)	PDI[a]
P1	$PDGu_p(6.7)$-b-$PBLK_p(13.3)$	$PDGu_p(6)$-b-$PBLK_p(14)$	6913	6519	1.10
P2	$PDGu_p(8)$-b-$PBLK_p(12)$	$PDGu_p(8)$-b-$PBLK_p(12)$	7151	6992	1.09
P3	$PDGu_p(10)$-b-$PBLK_p(10)$	$PDGu_p(10)$-b-$PBLK_p(10)$	7518	7398	1.11
P4	$PDGu_p(12)$-b-$PBLK_p(8)$	$PDGu_p(12)$-b-$PBLK_p(8)$	7884	7758	1.12
P5	$PDGu_p(13.3)$-b-$PBLK_p(6.7)$	$PDGu_p(14)$-b-$PBLK_p(6)$	8122	8074	1.13
P6	$PDGu_p(20)$	$PDGu_p(20)$	9350	8278	1.08

Supplementary Table 3. In vivo toxicity of **PDGu(7)-*b*-PBLK(13)**

Biomarkers	10 mg kg^{-1} daily										
	t=0 day		t=1 day			t=4 day			t=7 day		
	Mean	SD	Mean	SD	p value	Mean	SD	p value	Mean	SD	p value
TP	50.98	1.32	49.74	2.37	0.6035	51.76	3.67	0.6954	51.66	2.05	0.5238
ALB	29.42	2.58	28.34	2.21	0.8766	29.06	5.44	0.9129	29.34	2.12	0.8889
GLO	21.56	1.65	21.40	1.31	0.3588	22.70	1.82	0.4349	22.32	1.81	0.6905
TBIL	2.89	0.90	3.19	0.65	0.4717	2.40	0.21	0.3633	3.342	1.06	0.5476
ALT	44.80	6.57	44.00	7.04	0.6037	40.80	4.92	0.4118	42.8	5.26	0.5476
AST	75.60	13.01	75.40	7.37	0.6929	81.80	11.95	0.3036	71.6	9.40	0.6905
BUN	9.43	2.05	9.72	0.46	0.2214	9.05	0.67	0.6773	9.504	0.86	0.7857
CRE	55.20	13.22	61.60	7.70	0.6545	58.40	14.96	0.7927	55.6	22.23	0.8413
GLU	7.94	0.89	7.24	0.55	0.2391	6.38	0.41	0.0625	7.816	1.02	>0.9999
Na+	6.57	0.62	6.13	0.94	0.5808	6.48	0.51	>0.9999	5.986	0.37	0.0952
K+	142.00	4.74	139.60	11.24	0.0594	140.80	3.56	0.7500	145.2	4.02	0.3571

p values were calculated using one-way ANOVA followed by Dunnett multiple comparison test.

Supplementary Note

Circular dichroism study shows that the homocationic **PBLK(20)** transitions from a random coil to a left-handed helix. In water, the block copolymer **PDGu(7)-*b*-PBLK(13)** (Supplementary Figure 33c) likely adopts a helix-coil structure due to the sugar-cationic block[7,8] (Supplementary Figure 33a, b). However, in the presence of anionic liposomes of POPG which mimics bacterial membrane, **PBLK(20)** transforms to a left-handed helix conformation as indicated by a pronounced minimum at 213 nm, a zero crossing at 203 nm and a maximum at 196 nm (Supplementary Figure 33b).[9,10] In the presence of anionic liposomes, **PDGu(7)-*b*-PBLK(13)** copolymer also shows a more pronounced left-handed helical structure compared to that in water and it is likely that the cationic block transitions to the left-handed helical structure (Supplementary Figure 33c). However, when challenged with zwitterionic liposomes of POPC, which mimics mammalian cell surfaces, both **PBLK(20)** and **PDGu(7)-*b*-PBLK(13)** show no secondary structure transition.

To verify that the appropriate peptide:lipid (P:L) conditions were used, we further conducted the CD measurements at low (5:1) to high (around 1:80) P:L ratios while keeping the peptide concentration constant (Supplementary Figure 33d-f). The CD signal increases as the P:L ratio increases, but it plateaus at the charge neutralization ratio and beyond; the charge neutralization P:L ratios for the homocationic **PBLK(20)** and the copolymer **PDGu(7)-*b*-PBLK(13)** are 1:20 and 1:13 respectively. Hence our chosen P:L ratios of 1:79 and 1:89 for CD studies in Supplementary Figure 33a-c are beyond the saturation ratio for both charged polymers.

Simulation parameters

All Molecular Dynamics (MD) simulations were performed by Gromacs 4.6.3 package[11]. The CHARMM general force field[12] and CHARMM36 lipid force field[13] were used to characterize the beta-peptide sequence and the lipid molecules, respectively. During the simulation, a leap-frog algorithm was used to integrate Newton's equations of motion, and the time step was set to 2fs. The counter ions, sodium and chlorine were added to TIP3P water[14] model to neutralize the system and

reach a concentration of 0.15M physiological salt solution. All the covalent bonds in solutes and solvents were constrained by the LINCS algorithm[15] and the SETTLE algorithm[16], respectively. The cut-off for the short range electrostatics and Lennard-Jones interactions were both set to 1.2 nm and the particle mesh Ewald (PME)[17] algorithm was used to calculate the long range electrostatic interactions. Before the production simulations, energy minimization of the system was done using the steepest descent algorithm, and then the system was equilibrated by 100 ps simulation in NVT ensemble and 100 ps simulation in NPT ensemble with position restraint of the peptide. The reference temperature and pressure were set to 310K and 1 bar respectively, and the Nose-Hoover method[18] and the semi-isotropic Parrinello-Rahman method[19] (isotropic Parrinello-Rahman method for systems without membrane) were applied for temperature coupling and pressure coupling. All the visualization work was done by PyMol, version 1.5.0.3.

The well-tempered meta-dynamics simulations were performed with the help of the PLUMED 2.1.3 plug-in[20]. The system went through the same procedures as described above before the production sampling. We chose the number of the backbone H-bonds of the PBLK as a biased collective variable (CV) to enhance the production sampling. This CV is defined by the following switching function:

$$S_{ij}(t) = \frac{1 - (r_{ij}(t)/r_0)^n}{1 - (r_{ij}(t)/r_0)^m} \tag{1}$$

where $r_{ij}(t)$ gives the instantaneous distance between CV atom i and atom j, $r_0 = 0.25\,nm$ defines the distance cutoff for H-bonds, and n and m are 8 and 12 respectively. The Gaussian-width for H-bonds was set as 0.5. The initial Gaussian height was set to 2 kJ mol^{-1} and the bias factor was set to 8. The Gaussian deposition was performed every 1 ps.

Validity of the force field parameters for beta-peptide

Rathore *et al.* developed parameters for beta-peptide modelling based on the similar chemical structures available in standard CHARMM force field[21]. The 1-4 Coulomb interactions of the beta-peptide in their simulation is scaled down by a factor of 0.4. However, this scaled-down factor is not compatible with the lipid model used in our

simulation system, which is described by the CHARMM 36 lipid force field with a scaling factor of 1. Thus we didn't use their parameters for compatibility reasons. In our simulation, the beta-peptide parameters are created based on similar structures that are available in the CHARMM general force field. The backbone dihedral angles φ, θ, and ψ of the beta-peptide, as depicted by a typical beta-peptide in Supplementary Figure 34a, are described by CG2O1-NG2S1-CG311-CG321, NG2S1-CG311-CG321-CG2O1 and CG311-CG321-CG2O1-NG2S1 in the CHARMM general force field, respectively, where the CG2O1 is the carbonyl carbon, NG2S1 is the peptide nitrogen, CG311 is the aliphatic carbon in CH group, and CG321 is the aliphatic carbon in CH2 group. These parameters, according to the annotation from the force field, are obtained from the alanine dipeptide parameters, alkane parameters, and alanine dipeptide parameters, respectively.

To further confirm the validity of our parameters, additional benchmark simulations were performed with two reported beta-peptide sequences, whose helical structures had already been proven. They are (i) sequence 1 (seq1): β-Glu-β-Leu-β-homoornithine-β-Phe-β-Leu-β-Asp-β-Phe-β-Leu-β-homoornithine-β-homoornithine-β-Leu-β-Asp[22], and (ii) sequence 2 (seq2): ACHC-β-Lys-β-Leu-ACHC-β-Lys-β-Leu-ACHC-β-Lys-β-Leu[21]. Parameters of both sequences were developed with the same strategy as used in the present study. Both simulations started from the helical structures, and the stability of the helices were monitored during 100 ns simulation in water. As depicted in Supplementary Figure 34b which shows the time evolution of the Root Mean Square Deviation (RMSD) of the peptide backbone atoms from the initial helical state (all less than 0.2 nm), both sequences are maintained as helices, suggesting that our parameters are able to characterize the helical structure of these beta-peptides.

Verification of the helical structure of PBLK on the membrane:

Experimental data indicate that the PBLK could form a helical structure in the environment of the negatively charged membrane. Before further investigating its interaction with the membrane, it is necessary to verify this helical structure of the PBLK first. The PBLK sequence we used in the simulation contains 20 protonated units.

Instead of simulating the spontaneous formation of the helix from the random coil structure, which usually requires a large time scale of simulation, we started our simulation from well-defined helical structures and monitored their stability on the membrane. As reported, beta-peptides could form different helical structures including 14 helix, 12 helix, 10 helix and 8 helix[23,24], which are named based on the number of atoms comprising the H-bond ring. The well-defined helical structures were generated from coil structures by 5 ns simulation in water with the distance restraints to the specified H-bond donors and acceptors. Totally four common helical structures, including the left-handed 2.5_{12} helix (L12), the left-handed 3_{14} helix (L14), the right-handed 2.5_{12} helix (R12) and the right-handed 3_{14} helix (R14) were built by constructing the intramolecular H-bonds between the amide groups and carbonyl groups on the peptide backbone, more specifically, the amide groups of residue i and carbonyl groups of residue i-3 for 2.5_{12} helix, and the amide groups of residue i and carbonyl groups of residue i+2 for 3_{14} helix. The left-handed or right-handed in the nomenclature refers to the handedness of the helix, while 2.5/3, and 12/14 indicate the number of residues in one helical turn and the number of atoms comprising the H-bond ring, respectively. After that, each helix was placed above a membrane model lying in the xy-plane. The orientation of the helices are parallel to the membrane surface and their center of mass distance (z-component) are around 4.5 nm. These configurations were simulated freely for 10 ns. The membrane model employed, which mimics the cytoplasmic cell membrane of *Staphylococcus aureus*, is a mixture of 1,1'-palmitoyl-2,2'–vacenoyl cardiolipin (PVCL2) and 3-palmitoyl-2-oleoyl-D-glycero-1-phosphatidylglycerol (POPG) in the molar ratio of 42:58, as described in Epand and Epand[25]. The membrane was equilibrated for 200 ns before the simulation. The number of intramolecular H-bonds of each helix are shown in Supplementary Figure 34c, and only the L14 helix is stable. These results indicate that the protonated PBLK forms L14 helix on the negatively charged membrane.

The necessity of the negatively charged membrane in maintaining the L14 helix of the PBLK:

Using the same configuration, the stability of the protonated L14 helix on the membrane was further confirmed by 200 ns * 3 repeats with randomly assigned initial velocities of the system. And for comparison, the stabilities of the protonated and deprotonated (without side chain charges) L14 helixes in water environment were also studied by 200 ns * 3 repeats. The protonated L14 helix shows high stability on membrane since its intramolecular H-bonds are well-maintained during the whole simulation process (Supplementary Figure 34d, Protonated L14 on the membrane), but prefers coil rather than helical structure in water (Supplementary Figure 34d, Protonated L14 in water). This disruption of the helix in water is a consequence of electrostatic repulsion among the PBLK side chains: the stability is restored once the charges on the side chains are removed (Supplementary Figure 34d, Deprotonated L14 in water). When binding to the negatively charged membrane, the positive charges of the PBLK are neutralized by the environment, thus the electrostatic repulsion is greatly reduced and the stability of the helix is strengthened. These results support the hypothesis that the bacterial membrane environment functions as a charge-neutralizer in folding the PBLK.

The transition between different folding states of the peptide is usually not observable by the conventional MD simulations in a short time range. The system is easily trapped by the separated local minima on the free energy surface, making sufficient sampling unaffordable. In meta-dynamics, the selected CVs evolve in response to a biased potential composed by the real potential and a history-dependent bias. The history-dependent bias is a sum of Gaussians which center on the visited CV values, thus pushing the system to visit the less favored region and enhance the sampling. In this study, the folding free energy profile of the protonated PBLK under water or membrane environment is investigated by 200 ns well-tempered meta-dynamics simulations. The simulation starts from the well-defined L14 helix and the number of intramolecular H-bonds is selected as a CV. The Gaussian height stays as a small value in at least the last 50 ns simulation in both systems (Supplementary Figure 34e), indicating the convergence of our simulation. The free energy profile in water environment shows a small free energy barrier between fully-folded state and non-folded states. In contrast,

the free energy profile in the membrane environment is greatly modified by electrostatic attraction between the PBLK and the anionic lipids; the fully-folded state is separated by an energy barrier larger than 30 kJ mol^{-1} from the non-folded state in the membrane environment (Supplementary Figure 34f, g). As a consequence, the helical structure exists stably in the membrane environment but in the water environment eventually transforms into a coil. These results give explanations to the thermodynamic behaviors of the protonated PBLK sequence under different environment and prove the necessity of the anionic membrane in inducing the PBLK helix.

The interaction between PDGu(7)-*b*-PBLK(13) and negatively charged membrane: A helical model of **PDGu(7)-*b*-PBLK(13)** was simulated for 200 ns * 5 repeats in the membrane environment (Supplementary Figure 34h). This helix model was a combination of two well-defined helixes, in which the **PBLK** block (13 units) was an L14 helix and the **PDGu** block (7 units) was also a left-handed helix obtained from a short simulation of an optimized structure in gas phase[5]. Although these two helixes are both left-handed, they are structurally different from each other: one rise in the **PBLK** helix contains 3 residues with a height of 0.476±0.014 nm, while that in the **PDGu** helix has 3.5 residues with a height of 0.677±0.021 nm. Initially, the **PDGu(7)-*b*-PBLK(13)** was placed above the membrane with the z-component of the distance between the centers of masses set to be around 4.5 nm; the central axis of the helix was placed parallel to the y axis for repeats 1 to 3, x axis for repeat 4 or diagonal of the xy plane for repeat 5 (Supplementary Figure 34h shows one of the initial configurations). Repeats 1 to 3 were performed with same initial configuration but randomly assigned initial velocities. In all the repeats, the center of mass distance between peptide and membrane quickly decreased to around 2.5 nm after **PDGu(7)-*b*-PBLK(13)** attached to the surface of the membrane during the simulation (Supplementary Figure 34i).

The driving force during the binding of **PDGu(7)-*b*-PBLK(13)** to membrane was mainly provided by the **PBLK** block and the **PDGu** block protrudes into the water-membrane interface because of its weaker binding to the membrane than the **PBLK** block (Supplementary Figure 34h). In the border between the **PDGu** and the **PBLK**

segments, the DGu units form H-bonds with the carbonyl groups of the BLK units, using their –OH groups on C3 as the H-bond donors (Supplementary Figure 34j). Consequently, the **PDGu(7)-*b*-PBLK(13)** exists as one continuous helix during the whole binding process, as indicated by the small RMSD value of its heavy atoms with respect to those of the initial configuration (Supplementary Figure 34k).

In vivo murine systemic toxicity (intravenous injection) study

Clinically significant biomarkers were recorded before, and 24 hours, 4 days and 7 days after the first injection and data are expressed as mean ± standard deviation in Table 3. Alanine transaminase (ALT) level, aspartate transaminase (AST) level and their ratio (AST/ALT) are clinically significant biomarkers for hepatotoxicity. AST and ALT are enzymes normally contained in liver cells. Upon liver damage, the enzymes are released into blood and an increase of the enzyme level can be directly related to the extent of tissue damage[26]. TBIL (total bilirubin) measures the total amount of bilirubin (both conjugated and unconjugated with sugar) in the serum. Bilirubin is a catalytic product during the destruction of aged red blood cells, and it is processed by the liver for its elimination from the body. Possible causes of high TBIL include liver damage and elevated red blood cell hemolysis[27]. Serum creatinine (CRE) is a muscle metabolic byproduct and is cleared in the kidney. Elevated creatinine level is an indicator of kidney damage that leads to reduced clearance of creatinine. Blood glucose (GLU), blood urea nitrogen (BUN), potassium and sodium ion concentrations are other common biomarkers for kidney function and the electrolyte balance in the blood.

For histopathology studies, mice were sacrificed at 48 hours post last injection, and histological analysis was performed on the tissues obtained from their harvested organs (liver, kidney, and spleen). The sections were examined by a pathologist and no pathological changes were detected in comparison of tissue sections of treated mice with that of the controls. These results provide evidence that the prepared formulation has negligible acute tissue toxicity.

Supplementary References

1 Guichard, G., Abele, S. & Seebach, D. Preparation of *N*-Fmoc-Protected *β*[2]-and *β*[3]-Amino Acids and their use as building blocks for the solid-phase synthesis of *β*-peptides. *Helv. Chim. Acta.* **81**, 187-206 (1998).

2 Huang, H., Iwasawa, N. & Mukaiyama, T. A convenient method for the construction of *β*-lactam compounds from *β*-amino acids using 2-chloro-1-methylpyridinium iodide as condensing reagent. *Chem. Lett.* **13**, 1465-1466 (1984).

3 Chmielewski, M. & Kałuza, Z. [2+2] Cycloaddition of trichloroacetyl isocyanate to glycals. *Carbohydr. Res.* **167**, 143-152 (1987).

4 Liu, R. *et al.* Tuning the biological activity profile of antibacterial polymers via subunit substitution pattern. *Journal of the American Chemical Society* **136**, 4410-4418 (2014).

5 Dane, E. L. & Grinstaff, M. W. Poly-amido-saccharides: synthesis via anionic polymerization of a β-lactam sugar monomer. *Journal of the American Chemical Society* **134**, 16255-16264 (2012).

6 Dane, E. L., Chin, S. L. & Grinstaff, M. W. Synthetic enantiopure carbohydrate polymers that are highly soluble in water and noncytotoxic. *ACS Macro Lett.* **2**, 887-890 (2013).

7 Chin, S. L. *et al.* Combined molecular dynamics simulations and experimental studies of the structure and dynamics of poly-amido-saccharides. *Journal of the American Chemical Society* **138**, 6532-6540 (2016).

8 Abele, S., Guichard, G. & Seebach, D. (S)-β3-Homolysine-and (S)-β3-Homoserine-Containing β-Peptides: CD Spectra in Aqueous Solution. *Helvetica chimica acta* **81**, 2141-2156 (1998).

9 Gardiner, J. *et al.* β-Peptide Conjugates: Syntheses and CD and NMR Investigations of β/α-Chimeric Peptides, of a DPA-β-Decapeptide, and of a PEGylated β-Heptapeptide. *Helvetica Chimica Acta* **92**, 2698-2721 (2009).

10 Kritzer, J. A. *et al.* Relationship between side chain structure and 14-helix stability of β3-peptides in water. *Journal of the American Chemical Society* **127**, 167-178 (2005).

11 Berendsen, H. J. C., Spoel, D. v. d. & Drunen, R. v. GROMACS: a message passing parallel molecular dynamics implementation. *Comp. Phys. Comm.* **95**, 43-56 (1995).

12 Vanommeslaeghe, K. *et al.* CHARMM general force field: a force field for drug-like molecules compatible with the CHARMM all-atom additive biological force fields. *Journal of Computational Chemistry* **31**, 671-690 (2010).

13 Klauda, J. B. *et al.* Update of the CHARMM All-Atom Additive Force Field for Lipids: Validation on Six lipid Types. *J. Phys. Chem. B* **114**, 7830-7843 (2010).

14 Jorgensen, W. L., Chandrasekhar, J., Madura, J. D., Impey, R. W. & Klein, M. L. Comparison of simple potential functions for simulating liquid water. *The Journal of Chemical Physics* **79**, 926, doi:10.1063/1.445869 (1983).

15 Hess, B., Bekker, H., Berendsen, H. J. C. & Fraaije, J. G. E. M. LINCS: a linear constraint solver for molecular simulations. *J. Comput. Chem.* **18**, 1463-1472 (1997).

16 Miyamoto, S. & Kollman, P. A. SETTLE: An Analytical Version of the SHAKE and RATTLE Algorithm for Rigid Water Models. *J. Comput. Chem.* **13**, 952-962 (1992).

17 Essmann, U. *et al.* A smooth particle mesh Ewald method. *J. Chem. Phys.* **103**, 8577-8593 (1995).

18 Hoover, W. G. Canonical dynamics: Equilibrium phase-space distributions. *Phys. Rev. A* **31**, 1695-1697, doi:10.1103/PhysRevA.31.1695 (1985).

19 Parrinello, M. & Rahman, A. Crystal Structure and Pair Potentials: A Molecular-Dynamics Study. *Phys. Rev. Lett.* **45**, 1196-1199, doi:10.1103/PhysRevLett.45.1196 (1980).

20 Bonomi, M. *et al.* PLUMED: A protable plugin for free-energy calculations with molecular dynamics. *Comp. Phys. Comm.* **180**, 1961-1972 (2009).

21 Rathore, N., Gellman, S. H. & de Pablo, J. J. Thermodynamic stability of beta-peptide helices and the role of cyclic residues. *Biophys J* **91**, 3425-3435, doi:10.1529/biophysj.106.084491 (2006).

22 Daniels, D. S., Petersson, E. J., Qiu, J. X. & Schepartz, A. High-Resolution Structure of a beta-Peptide Bundle. *J Am Chem Soc* **129**, 1532-1533 (2007).

23 Cheng, R. P., Gellman, S. H. & DeGrado, W. F. beta-Peptides: From Structure to Function. *Chem. Rev.* **101**, 3219-3232 (2001).

24 Christofferson, A. J. *et al.* Identifying the Coiled-Coil Triple Helix Structure of beta-Peptide Nanofibers at Atomic Resolution. *ACS Nano* **12**, 9101-9109, doi:10.1021/acsnano.8b03131 (2018).

25 Epand, R. M. & Epand, R. F. Bacterial membrane lipids in the action of antimicrobial agents. *J Pept Sci* **17**, 298-305, doi:10.1002/psc.1319 (2011).

26 Lin, C.-F. *et al.* Liver injury caused by antibodies against dengue virus nonstructural protein 1 in a murine model. *Lab. Invest.* **88**, 1079 (2008).

27 Roberton, N. & Rennie, J. M. *Rennie & Roberton's Textbook of Neonatology*. (Elsevier, 2012).
