# Supplementary material for: Enantiomeric glycosylated cationic block co-beta-peptides eradicate Staphylococcus aureus biofilms and antibiotic-tolerant persisters
Source: Nat Commun. 2019 Oct 21;10:4792. doi: 10.1038/s41467-019-12702-8 (PMC6803644; doi:10.1038/s41467-019-12702-8)
Supplement: Supplementary file 3 — Reporting Summary [file 41467_2019_12702_MOESM3_ESM.pdf]

nature research

Corresponding author(s): Kevin Pethe and Mary B. Chan-Park

Last updated by author(s): Sep 5, 2019

Reporting Summary

Nature Research wishes to improve the reproducibility of the work that we publish. This form provides structure for consistency and transparency in reporting. For further information on Nature Research policies, see Authors & Referees and the Editorial Policy Checklist.

Statistics

For all statistical analyses, confirm that the following items are present in the figure legend, table legend, main text, or Methods section.

n/a	Confirmed	
☐	☒	The exact sample size (*n*) for each experimental group/condition, given as a discrete number and unit of measurement
☐	☒	A statement on whether measurements were taken from distinct samples or whether the same sample was measured repeatedly
☐	☒	The statistical test(s) used AND whether they are one- or two-sided<br>*Only common tests should be described solely by name; describe more complex techniques in the Methods section.*
☒	☐	A description of all covariates tested
☒	☐	A description of any assumptions or corrections, such as tests of normality and adjustment for multiple comparisons
☐	☒	A full description of the statistical parameters including central tendency (e.g. means) or other basic estimates (e.g. regression coefficient) AND variation (e.g. standard deviation) or associated estimates of uncertainty (e.g. confidence intervals)
☐	☒	For null hypothesis testing, the test statistic (e.g. *F*, *t*, *r*) with confidence intervals, effect sizes, degrees of freedom and *P* value noted<br>*Give P values as exact values whenever suitable.*
☒	☐	For Bayesian analysis, information on the choice of priors and Markov chain Monte Carlo settings
☒	☐	For hierarchical and complex designs, identification of the appropriate level for tests and full reporting of outcomes
☒	☐	Estimates of effect sizes (e.g. Cohen's *d*, Pearson's *r*), indicating how they were calculated

Our web collection on statistics for biologists contains articles on many of the points above.

Software and code

Policy information about availability of computer code

Data collection	No previously unreported custom computer code or algorithm were used in this study.
Data analysis	Graphpad Prism 7, FlowJo V10, ImageJ Version 1.51

For manuscripts utilizing custom algorithms or software that are central to the research but not yet described in published literature, software must be made available to editors/reviewers. We strongly encourage code deposition in a community repository (e.g. GitHub). See the Nature Research guidelines for submitting code & software for further information.

Data

Policy information about availability of data

All manuscripts must include a data availability statement. This statement should provide the following information, where applicable:

- Accession codes, unique identifiers, or web links for publicly available datasets
- A list of figures that have associated raw data
- A description of any restrictions on data availability

The data that support the findings of this study are available from the authors on reasonable request, see author contributions for specific data sets. The source data underlying Figs. 4e, 5a-f, 6a, b, d-g, Supplementary Fig. 16a-d and Supplementary Table 3 are provided in the Source Data file.

Field-specific reporting

Please select the one below that is the best fit for your research. If you are not sure, read the appropriate sections before making your selection.

☒ Life sciences ☐ Behavioural & social sciences ☐ Ecological, evolutionary & environmental sciences

Life sciences study design

All studies must disclose on these points even when the disclosure is negative.

Sample size	Sample size was not predetermined using a statistical method. Sample size was determined to be adequate based on the magnitude and consistency of measurable differences between groups.
Data exclusions	The authors declare no data exclusion.
Replication	The in vitro and in vivo data was reproduced with at least two samples.
Randomization	No randomization was used in this study.
Blinding	No blinding was used in this study.

Reporting for specific materials, systems and methods

We require information from authors about some types of materials, experimental systems and methods used in many studies. Here, indicate whether each material, system or method listed is relevant to your study. If you are not sure if a list item applies to your research, read the appropriate section before selecting a response.

Materials & experimental systems

n/a	Involved in the study	
☒	☐	Antibodies
☐	☒	Eukaryotic cell lines
☒	☐	Palaeontology
☐	☒	Animals and other organisms
☐	☒	Human research participants
☒	☐	Clinical data

Methods

n/a	Involved in the study	
☒	☐	ChIP-seq
☐	☒	Flow cytometry
☒	☐	MRI-based neuroimaging

Eukaryotic cell lines

Policy information about cell lines

Cell line source(s)	ATCC
Authentication	The cell line was authenticated by STR profiling.
Mycoplasma contamination	No mycoplasma contamination.
Commonly misidentified lines (See ICLAC register)	No commonly misidentified lines.

Animals and other organisms

Policy information about studies involving animals; ARRIVE guidelines recommended for reporting animal research

Laboratory animals	8 week female C57BL6 mice (weight ~20 g) were used for the in vivo efficacy study in wound models. 8 week female BALB/c mice (weight 18-22g) were used for in vivo systemic toxicity study and acute systemic efficacy study. 6 week female ICR mice (weight 20-25g) were used for in vivo efficacy study in thigh model.
Wild animals	No wild animals were used in this study.
Field-collected samples	No field-collected samples were involved in this study.
Ethics oversight	All the animal protocols were reviewed and approved by the Animal Ethics and Welfare Committee (AEWC) of Ningbo University.

Note that full information on the approval of the study protocol must also be provided in the manuscript.

Human research participants

Policy information about studies involving human research participants

Population characteristics	Human blood for hemolysis study was collected from a male donor aged 23 (Chinese). Human skin samples for ex vivo wound study were from female donors aged 66, 72 and 67 respectively (Caucasian).
Recruitment	Human blood donor was recruited under the approved protocol (IRB-2015-03-040) with informed consent. Human skin samples were purchased from Biopredic International and informed consent was given in accordance with French law and ethical principles.
Ethics oversight	Nanyang Technological University

Note that full information on the approval of the study protocol must also be provided in the manuscript.

Flow Cytometry

Plots

Confirm that:

- [x] The axis labels state the marker and fluorochrome used (e.g. CD4-FITC).
- [x] The axis scales are clearly visible. Include numbers along axes only for bottom left plot of group (a 'group' is an analysis of identical markers).
- [ ] All plots are contour plots with outliers or pseudocolor plots.
- [x] A numerical value for number of cells or percentage (with statistics) is provided.

Methodology

Sample preparation	Detailed sample preparation procedures were provided in the main and supplementary files.
Instrument	BD Accuri C6 plus
Software	BD Accuri C6 Plus software for data collection and FlowJo V10 for data analysis.
Cell population abundance	Not applicable. Flow cytometry was used for bacteria sample analysis only.
Gating strategy	Bacteria without dye and heat-killed bacteria were used as negative and positive controls respectively to differentiate the population.

- [ ] Tick this box to confirm that a figure exemplifying the gating strategy is provided in the Supplementary Information.
